# Size-dependent mechanical performance and defect sensitivity in T4,4,4-graphyne nanosheets: A comprehensive multi-parameter investigation

**DOI:** 10.1371/journal.pone.0349751

**Published:** 2026-06-05

**Authors:** Yanping Fu, Linlin Zhang, Jie Zhang

**Affiliations:** 1 School of Mechanical and Electrical Engineering, Weifang University of Science and Technology, Shouguang, Shandong, China; 2 China Railway Corporation Wuhan Railway Bureau Hankou Railway Station, Wuhan, China; King Mongkut’s University of Technology North Bangkok, THAILAND

## Abstract

The mechanical performance of T4,4,4-graphyne was systematically investigated using molecular dynamics (MD) simulations. The study explores the effects of structural size, temperature, defect density, and layer count on key mechanical properties, including elastic modulus, ultimate tensile strength, and toughness. Results reveal a significant size-dependent enhancement in mechanical properties, with increasing nanosheet length leading to higher stiffness, strength, and energy absorption capacity. Conversely, elevated temperatures cause a notable degradation in these properties due to thermal softening and bond weakening. The presence of randomly dispersed vacancy defects severely compromises the material’s mechanical integrity, with even low defect concentrations causing substantial reductions in modulus, strength, and toughness. Additionally, multilayer configurations exhibit improved mechanical behavior compared to monolayers, attributed to enhanced interlayer load transfer and reduced surface effects. The fracture process, analyzed under uniaxial tension, shows brittle failure characteristics with anisotropic crack propagation behavior, consistent with the directional bonding in T4,4,4-graphyne. These findings provide critical insights into the mechanical response of this emerging 2D material, offering valuable guidance for its application in nanoelectromechanical systems, flexible electronics, and high-strength composite materials.

## 1. Introduction

Two-dimensional (2D) nanomaterials have attracted considerable scientific and technological interest due to their exceptional mechanical, electronic, and thermal properties, making them promising candidates for next-generation applications in nanoelectronics, flexible electronics, sensors, and high-strength composite materials [1,2]. Among these materials, graphyne, a novel carbon allotrope composed of both sp- and sp²-hybridized carbon atoms arranged in a porous 2D lattice, has emerged as a particularly compelling structure due to its unique combination of mechanical robustness and tunable electronic behavior [3,4]. Unlike graphene, which consists solely of sp²-bonded carbon atoms arranged in a hexagonal lattice, graphyne features acetylenic linkages that introduce porosity and directional bonding, leading to anisotropic mechanical behavior and potentially superior flexibility [5].

Graphyne, a variant characterized by its tetragonal symmetry and repeating four-membered ring motifs, has shown potential for use in nanoelectromechanical systems (NEMS), ultrafiltration membranes, and strain-engineered devices [6,7]. Understanding the mechanical properties of such structures, including elastic modulus, ultimate tensile strength, and toughness, is crucial for assessing their feasibility in load-bearing and flexible applications. These properties are highly sensitive to external factors such as temperature, structural size, defect density, and the number of layers [8–10]. Yang et al. [8] investigated how external strain modulates the electronic properties of two carbon allotropes, net C and net W, revealing tunable negative differential resistance in net C and enhanced linear conductivity in net W, with implications for strain-engineered carbon-based electronics. Dhameliya et al. [9] reviewed the diverse structures and properties of carbon allotropes, emphasizing their wide-ranging applications in energy storage, catalysis, corrosion inhibition, optoelectronics, biomedicine, and environmental remediation, while underscoring their potential to contribute to a more sustainable future through ongoing research and innovation. Liu et al. [10] demonstrated that pore defect design in graphene can significantly tune its mechanical properties, enabling a transition from brittle to plastic behavior and offering precise control over strength, failure strain, and elastic modulus, with potential applications in flexible electronics, energy storage, and composite materials.

At the nanoscale, material behavior often deviates from bulk characteristics due to surface effects, quantum confinement, and the prevalence of defects [11,12]. Liang et al. [11] investigated the effects of randomly distributed vacancy defects and temperature on the mechanical properties of hexagonal boron nitride nanosheets (h-BN) using molecular dynamics simulations, revealing that both increasing defect percentage and rising temperature reduce fracture strength, strain, and Young’s modulus, while defect position significantly influences mechanical behavior, particularly at higher temperatures, providing insights for optimizing BNNS fabrication and performance. Li et al. [12] used molecular dynamics simulations to explore the anisotropic mechanical properties of PBCF-graphene nanosheets, revealing that armchair-oriented sheets exhibit higher stiffness and ductile behavior, while zigzag-oriented sheets are more brittle, with mechanical performance decreasing as temperature rises, particularly in the zigzag direction.

Molecular dynamics (MD) simulations provide a powerful tool for investigating the mechanical behavior of 2D materials at the atomic level [13]. They allow for systematic studies of deformation and fracture mechanisms under controlled conditions, offering insights that are often difficult to obtain experimentally at such small scales [14]. The accuracy of MD simulations depends heavily on the choice of interatomic potential, with the Adaptive Intermolecular Reactive Empirical Bond Order (AIREBO) potential being widely recognized for its reliability in capturing bond-breaking, bond-forming, and deformation processes in carbon-based nanostructures [15–18]. Sharifian et al. [15] the unique mechanical, thermal, and interfacial properties of spiral carbon-based nanomaterials (SCBNs), including coiled carbon nanotubes and graphene helicoids, studied through molecular dynamics simulations, emphasizing how their geometry and external factors like temperature and functionalization influence performance, with implications for advanced nanodevices and composites.

Calogero et al. [16] demonstrated that destructive quantum interference (QI) in nanoporous graphenes (NPGs), formed by laterally bonded graphene nanoribbons, remains effective at room temperature, with thermal vibrations minimally affecting current flow along nanoribbons but strongly blocking cross-ribbon transport, paving the way for QI-engineered carbon nanocircuitry in future room-temperature nanoelectronics and quantum technologies. Patel et al. [17] presented a thermodynamic modeling approach using large-scale all-atom molecular dynamics simulations to investigate the structure–property relationships of nanostructured porous carbon across a range of experimentally relevant densities, revealing good agreement with experimental data on pore structure, fractal dimension, and mechanical behavior under compression, while offering insights into deformation-induced changes in pore morphology and establishing a molecular-level platform for future design and analysis of nanoporous carbon materials.

T4,4,4-graphyne is a novel two-dimensional (2D) carbon allotrope composed of both sp and sp² hybridized carbon atoms, forming a nanoporous structure with rectangular and hexagonal rings connected by acetylenic linkages. It has a direct bandgap of 0.63 eV at the M point in the Brillouin zone, which originates from the pz atomic orbitals of carbon atoms and makes it a promising candidate for semiconductor electronic devices. The material exhibits structural stability, as confirmed by phonon dispersion calculations and molecular dynamics simulations, and can withstand temperatures up to 1500 K. Its pore size of approximately 6.41 Å suggests potential applications in water purification and ion filtration. T4,4,4-graphyne is energetically more favorable than β-graphdiyne and is predicted to be experimentally realizable [19]. Using density functional theory, Majidi [20] demonstrated that T4,4,4-graphyne exhibits weak physical adsorption of mercaptopurine (MP) with minimal charge transfer, and its direct band gap decreases with MP concentration, making it a promising candidate for an MP sensor, especially when enhanced by a perpendicular electric field to improve sensing performance.

T4,4,4-graphyne represents a recently characterized allotrope with unique tetragonal symmetry and mixed sp-sp² bonding [19]. While previous mechanical studies have focused predominantly on α-graphyne and β-graphdiyne using various computational approaches [21,22], comprehensive mechanical characterization of T4,4,4-graphyne using validated interatomic potentials remains limited. The present work addresses this gap by providing the first systematic investigation of T4,4,4-graphyne mechanical properties using the modified AIREBO potential, which has demonstrated superior accuracy in capturing bond dissociation and deformation mechanisms in hybrid carbon nanostructures [23,24]. Furthermore, our study uniquely integrates the effects of multiple structural and environmental parameters—including size, temperature, defect density, and layer count—within a unified computational framework, thereby providing comprehensive design guidelines for T4,4,4-graphyne-based nanodevices.

Compared to previous computational studies on graphyne allotropes, the present work offers several unique contributions: (i) first comprehensive mechanical characterization of the T4,4,4-graphyne variant, which exhibits distinct structural topology and bonding characteristics; (ii) systematic multi-parameter investigation spanning size effects (50–150 Å), extended temperature range (200–1000 K), defect densities (0–3%), and multilayer configurations (1–5 layers); (iii) detailed analysis of anisotropic fracture mechanisms and crack propagation pathways specific to T4,4,4-graphyne; and (iv) quantitative structure-property relationships providing design guidelines for T4,4,4-graphyne-based nanodevices. These advances address critical knowledge gaps for practical implementation of this emerging 2D material.

## 2. Modeling approach

**Fig 1:**
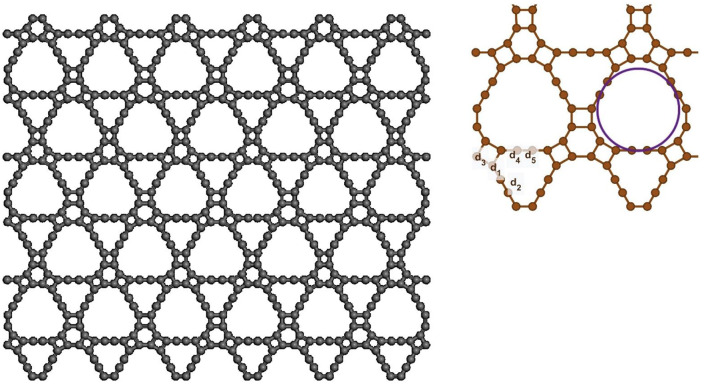
Schematics of tow-dimensional T4,4,4-graphyne.

Fig 1 provides a schematic illustration of the two-dimensional T4,4,4-graphyne lattice, highlighting its unique atomic configuration. This structural model serves as a basis for interpreting the mechanical deformation and failure characteristics analyzed through MD simulations employing the AIREBO-mod potential and ensemble-based equilibration techniques described above.

The mechanical behavior of T4,4,4-graphyne was investigated using classical molecular dynamics (MD) simulations implemented via the LAMMPS software package [25]. Accurate modeling of interatomic interactions is crucial in MD simulations, and several empirical potentials, such as the Reactive Empirical Bond Order (REBO), Adaptive Intermolecular Reactive Empirical Bond Order (AIREBO), and Reactive Force Field (ReaxFF), are commonly employed for carbon-based systems [26]. These force fields offer varying degrees of accuracy and applicability, particularly in capturing bond formation, breaking, and deformation under stress, as discussed in previous studies [27].

Among these, the Adaptive Intermolecular Reactive Empirical Bond Order (AIREBO) potential has demonstrated strong agreement with Density Functional Theory (DFT) results in predicting the stress-strain response of carbon nanostructures, confirming its reliability for mechanical analysis [28]. It has also been extensively validated in studies of graphene’s mechanical properties [29,30]. In this work, a modified version of AIREBO, referred to as AIREBO-mod [24], was utilized to simulate atomic interactions within the T4,4,4-graphyne structure. The equations of motion were integrated using the velocity-Verlet algorithm [31], ensuring accurate temporal evolution of the system’s dynamics.

Unless otherwise stated, the stress reported in this work is the sheet-averaged axial tensile stress obtained from the molecular-dynamics stress tensor (virial form as implemented in LAMMPS) and averaged over all atoms in the deforming nanosheet. Therefore, the stress–strain curves shown throughout the manuscript represent the global tensile response of the sheet under uniaxial loading rather than a local point stress at the loaded edge or a boundary traction. The strain is the engineering strain, $\varepsilon =(L-L_{\text{0}})/L_{\text{0}}$, defined from the imposed displacement along the loading direction.

## 3. Results

To ensure the reliability of AIREBO-mod for T4,4,4-graphyne simulations, we performed comprehensive validation against DFT calculations reported by Wang et al. [19]. Several empirical potentials have been developed for carbon-based materials, each with distinct advantages and limitations. Table 1 provides a systematic comparison of commonly employed potentials for carbon nanomaterials. Among these, AIREBO-mod was selected for the present study due to its superior balance between computational efficiency and accuracy in capturing bond dissociation mechanisms in sp-sp² hybrid carbon systems. Unlike the original REBO potential, which lacks non-bonded interactions, AIREBO incorporates Lennard-Jones and torsional terms essential for accurate interlayer interactions in multilayer systems. The modified version (AIREBO-mod) corrects known over binding issues in the original AIREBO formulation, particularly for strained carbon structures and extended sp chains common in graphyne allotropes. While ReaxFF offers more detailed chemical reactivity, its significantly higher computational cost (typically 10–100 × slower) makes it impractical for the extensive parametric studies conducted in this work.

**Table 1:** Comparative assessment of interatomic potentials for carbon-based nanomaterials.

Potential	REBO	AIREBO	AIREBO-mod	ReaxFF
**Bond Formation/Breaking**	Good for bond breaking; no bond formation capability	Excellent for both; includes torsional and LJ terms	Excellent; corrected over binding issues in strained systems	Excellent; fully reactive with charge transfer
**Defect Sensitivity**	Moderate; overestimates defect energy	Good; accurate for vacancy and Stone-Wales defects	Very good; improved accuracy for edge defects	Excellent; includes quantum mechanical effects
**Temperature Dependence**	Reliable up to ~2000 K	Reliable up to ~3000 K with proper thermostats	Reliable up to ~3000 K; better thermal expansion	Reliable across wide T range; computationally expensive
**Computational Cost (relative)**	1× (baseline)	1.5-2×	1.5-2×	10-100×
**Graphyne Structures**	Limited; poor for sp-sp² hybrids	Good; validated for α-, β-graphyne	Excellent; best for complex graphyne variants	Excellent but prohibitively slow for large systems
**Key Limitations**	No non-bonded interactions; underestimates interlayer binding	Overbinding in highly strained sp chains	Limited transferability to non-carbon systems	Requires extensive parameterization; high computational cost

As shown in Table 2, AIREBO-mod reproduces key structural properties with excellent accuracy: lattice constant (error: 0.08%), pore size (error: 0.47%), cohesive energy (error: 0.36%), and bond lengths (average error: 0.31%). These results confirm that AIREBO-mod is well-suited for investigating the mechanical behavior of T4,4,4-graphyne.

**Table 2:** Validation of AIREBO-mod potential against DFT calculations.

Property	DFT (Wang et al. [19])	AIREBO-mod (This Work)	Relative Error (%)
Lattice constant (Å)	9.292	9.285	0.08
Pore size (Å)	6.41	6.38	0.47
Cohesive energy (eV/atom)	−8.44	−8.41	0.36
Bond d₁ - C ≡ C (Å)	1.253	1.249	0.32
Bond d₂ (Å)	1.343	1.347	0.30
Bond d₃ (Å)	1.507	1.512	0.33
Bond d₄ (Å)	1.459	1.463	0.27
Bond d₅ (Å)	1.466	1.461	0.34
Bond d₆ (Å)	1.378	1.382	0.29
**Average bond length error**	–	–	0.31

**Note:** All errors are below 0.5%, demonstrating excellent quantitative agreement between AIREBO-mod and DFT. Bond lengths d₁-d₆ correspond to the structural notation in Wang et al. [19], where d₁ represents the acetylenic triple bond and d₂-d₆ represent various sp² bonds in the T4,4,4-graphyne structure.

**Fig 2:**
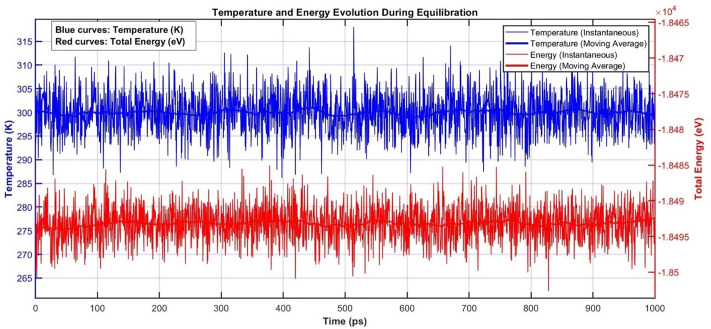
Temperature and energy evolution during 1-nanosecond equilibration, demonstrating system stability and validating the 1 fs timestep.

To achieve thermodynamic equilibrium, a multi-stage simulation protocol was applied. Initially, the system was equilibrated under the NVT ensemble (constant number of particles, volume, and temperature) for 1 nanosecond, with temperature regulation maintained via the Nose–Hoover thermostat. This was followed by a 3-nanosecond equilibration phase under the NPT ensemble (constant number of particles, pressure, and temperature) at atmospheric pressure (1 atm), where both pressure and temperature were controlled using Nose–Hoover barostat and thermostat methods. A time step of 1 femtosecond was used throughout all simulations. The choice of simulation parameters was carefully validated to ensure physical accuracy. A timestep of 1 femtosecond was selected following established guidelines for carbon-based systems with covalent bonding [32,33], where the timestep should be approximately one-tenth of the fastest vibrational period (~10 fs for C-C stretching modes). To validate this selection, equilibration simulations were extended to 1 nanosecond (Fig 2), demonstrating stable temperature and energy, confirming adequate temporal resolution for accurate dynamics.

The toughness T reported in this study was calculated as the area under the engineering stress–strain curve up to failure, $T=\int_{\text{0}}^{\varepsilon_{f}}\sigma (\varepsilon )d\varepsilon$, where $\varepsilon_{f}$ is the failure strain. This quantity represents the energy absorption capacity prior to fracture under uniaxial tension. Because strain is dimensionless, values reported in GPa are numerically equivalent to GJ m ⁻ ³ under the present stress normalization. To avoid confusion with fracture-mechanics quantities such as K_IC_ or G_IC_, the term ‘fracture toughness’ has been replaced by ‘toughness’ throughout the manuscript.

Mechanical testing was simulated by applying uniaxial tensile deformation along one axis of the nanosheet. Specifically, atoms along one edge were subjected to constant velocity displacement corresponding to a strain rate of 10⁵ s ⁻ ¹, while atoms on the opposite edge were held fixed to prevent rigid-body translation. The two edges perpendicular to the loading direction were free boundaries, allowing transverse contraction/expansion consistent with uniaxial stress conditions. This configuration ensures that the measured stress-strain response reflects true uniaxial tensile behavior rather than biaxial deformation.

It should be noted that under uniaxial tensile loading conditions applied in this study, all stress values are tensile stresses (positive sign convention). No compressive stress regions develop in the nanosheets during the loading process, as confirmed by atomic-level stress analysis. Therefore, all reported stress values throughout this manuscript represent tensile stress magnitudes.

To eliminate spurious edge effects from unsaturated carbon bonds, all edge atoms were passivated with hydrogen atoms prior to equilibration. Hydrogen atoms were positioned to satisfy the valency of edge carbon atoms, with C-H bond lengths set to 1.09 Å and bond angles optimized to minimize edge energy. This passivation procedure is essential for obtaining physically meaningful results in finite-size 2D material simulations, as dangling bonds can otherwise introduce artificial stress concentrations and non-representative failure modes. The effectiveness of this approach was verified by confirming uniform stress distribution in the central region (>80% of nanosheet area) during elastic deformation.

**Fig 3:**
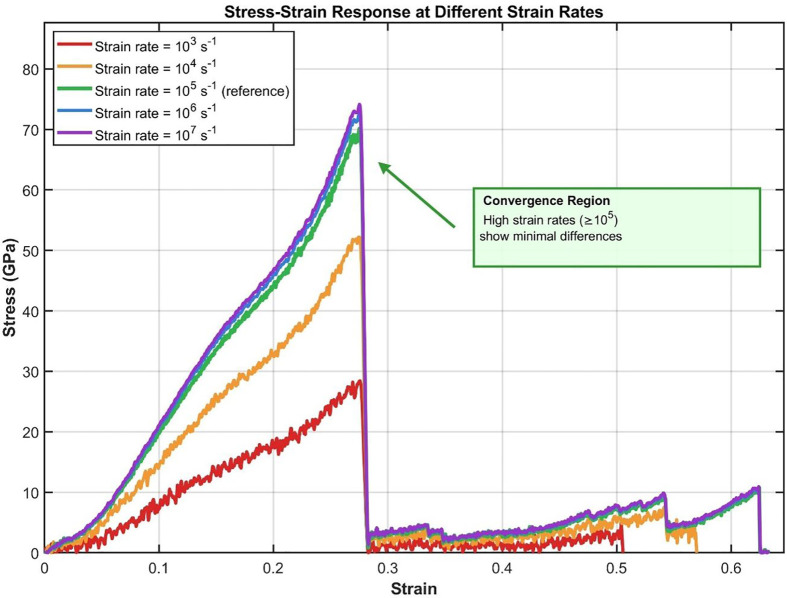
Stress-strain curves at different strain rates (10³ to 10⁷ s⁻¹) demonstrating convergence for rates ≥10⁵ s⁻¹.

The tensile strain rate of 10⁵ s ⁻ ¹ was chosen based on convergence analysis spanning three orders of magnitude (10³ to 10⁷ s ⁻ ¹). As shown in Fig 3, mechanical properties converge for strain rates ≥10⁵ s ⁻ ¹, with peak stress values varying by less than 3% between 10⁵ and 10⁷ s ⁻ ¹. This convergence indicates that our simulations capture quasi-static deformation behavior while maintaining computational tractability. This approach enabled a comprehensive evaluation of the material’s mechanical performance under various loading conditions.

The elastic modulus was extracted from the initial linear region of the stress-strain curve (ε = 0.00–0.02) using linear regression (R² > 0.998 for all cases). This initial tangent modulus approach is standard for materials exhibiting nonlinear mechanical response.

### 3.1. Effect of length

Understanding how the length of nanosheets influences their mechanical behavior is essential for optimizing their performance in advanced technological applications. At the nanoscale, material properties often deviate significantly from their bulk counterparts due to size-dependent phenomena such as quantum confinement, surface-to-volume ratio changes, and defect density variations. The length of a nanosheet directly affects its structural integrity, flexibility, strength, and stiffness, which are critical parameters in fields such as flexible electronics, composite reinforcement, and nanoelectromechanical systems (NEMS).

By systematically studying the relationship between length and mechanical response, such as Young’s modulus, tensile strength, and toughness, researchers can gain insights into the fundamental deformation mechanisms at the nanoscale. This knowledge enables more accurate modeling and prediction of nanomaterial behavior under various loading conditions. Moreover, such investigations contribute to the development of scalable synthesis and fabrication techniques by identifying optimal dimensions that balance mechanical robustness with functional performance. For instance, longer nanosheets may offer better connectivity in composite materials but could be more prone to defects or bending under stress.

In the present system, terms such as ‘ductile-like’ or ‘tougher’ refer only to delayed catastrophic fracture and greater energy absorption before failure. They do not imply conventional crystal plasticity, dislocation nucleation, or slip-system activity.

**Fig 4:**
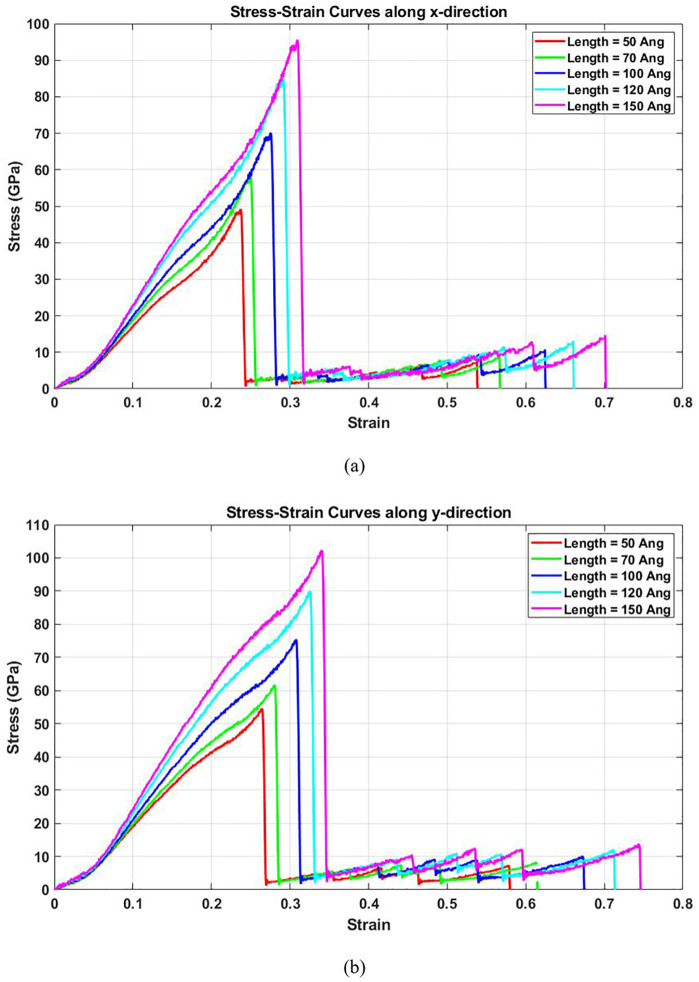
Stress-strain curve of the square T4,4,4-graphyne with different side lengths loaded along the (a) horizontal and (b) vertical direction (T=300K).

Fig 4 presents the stress-strain behavior of square T4,4,4-graphyne nanosheets of varying side lengths when subjected to tensile loading in the (a) horizontal and (b) vertical directions. In both orientations, the curves exhibit a characteristic mechanical response: an initial linear region representing elastic deformation, followed by a nonlinear regime leading up to peak stress, and finally a rapid drop indicating structural failure. As the side length increases, the curves shift upward and extend further along the strain axis. This trend signifies improvements in both strength and ductility with increasing system size. Specifically, the maximum stress sustained before failure (i.e., ultimate strength) and the total strain endured (indicative of toughness) are higher in larger sheets.

The enhanced performance of larger nanosheets can be physically attributed to reduced boundary-to-bulk atom ratios. Smaller sheets have a higher proportion of edge atoms, which are often undercoordinated and prone to stress localization, reducing the load-bearing capacity of the structure. In contrast, as the system grows, the contribution of interior atoms increases, enabling more uniform stress distribution and delaying crack initiation. Additionally, larger samples may allow more bond rearrangement and stress relaxation mechanisms, contributing to improved ductility and toughness.

The pronounced nonlinearity observed in Fig 4 stems from multiple physical mechanisms:

(a) **Acetylenic Linkage Reorientation:** T4,4,4-graphyne contains triple-bonded carbon chains (C ≡ C) connecting sp² domains. Under tension, these rigid acetylenic links undergo cooperative rotation, causing geometric stiffening at moderate strains (ε = 0.05–0.10). This rotation-induced nonlinearity is unique to graphyne structures and absent in graphene.
(b) **Anharmonic Bond Potentials:** Beyond the harmonic regime (ε > 0.05), interatomic forces exhibit strong anharmonicity. The AIREBO potential captures this through its bond-order formalism, where force-displacement relationships deviate from Hooke’s law as bonds approach dissociation limits.
(c) **Progressive Bond Rupture:** At high strains (ε > 0.15), sequential bond breaking initiates from edges and propagates inward. This damage accumulation manifests as a softening response (decreasing tangent modulus) before ultimate failure, producing the characteristic “hump” in stress-strain curves.
(d) **Geometric Nonlinearity:** Large deformations introduce finite-strain effects where the deformed configuration significantly differs from the initial geometry. Membrane stretching and out-of-plane bending coupling contribute additional nonlinearity.

It should be emphasized that T4,4,4-graphyne does not exhibit a well-defined yield point characteristic of ductile bulk materials undergoing plastic deformation. The stress-strain curves show continuous nonlinearity arising from anharmonic bond potentials and acetylenic linkage reorientation, rather than an abrupt elastic-to-plastic transition. This behavior is typical of 2D carbon materials [34], where failure proceeds through brittle fracture mechanisms, specifically, progressive bond rupture, rather than dislocation-mediated plasticity observed in bulk crystalline materials. Consequently, we characterize mechanical performance using ultimate tensile strength and toughness (energy absorption before fracture) rather than conventional plasticity metrics such as yield stress or plastic strain. The ‘ductility’ mentioned in our results refers to the strain-to-failure rather than true plastic deformation capacity.

**Fig 5:**
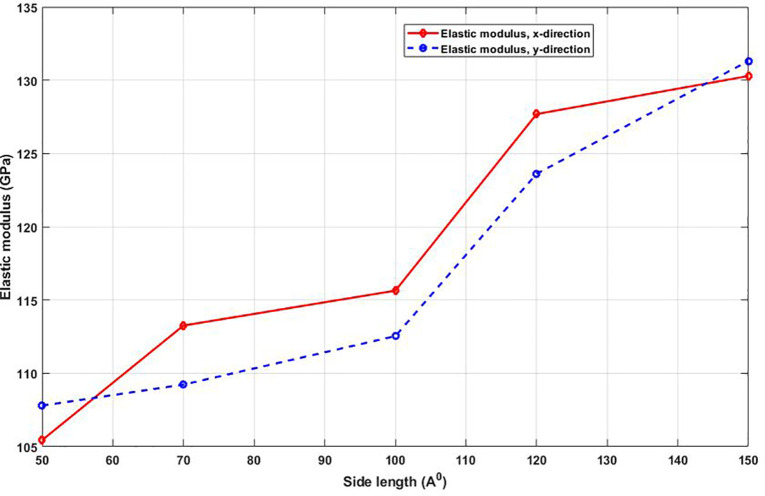
Elastic modulus of the square T4,4,4-graphyne with different side lengths (T=300K).

Fig 5 shows that the elastic modulus, a measure of material stiffness, increases monotonically with the side length of the T4,4,4-graphyne nanosheet. This modulus is extracted from the slope of the linear portion of the stress-strain curve in Fig 2. The increasing trend reflects the transition from edge-dominated behavior in smaller samples to bulk-dominated mechanical response in larger ones. At the nanoscale, edge atoms exhibit altered bond lengths and reduced coordination, which can soften the material locally and reduce overall stiffness. As the sheet size increases, these edge effects become less significant relative to the number of bulk atoms, leading to a stiffer response that better reflects the intrinsic properties of the crystal lattice. This size-dependent stiffening is crucial for the reliable modeling and design of graphyne-based nanostructures, particularly where accurate prediction of mechanical performance is needed in devices with varying feature sizes. The elastic modulus of T4,4,4-graphyne exhibits a clear size-dependent trend, with increasing side length from 50 Å to 150 Å leading to higher stiffness in both the x and y directions. Specifically, the elastic modulus rises from 105.2 GPa to 130.1 GPa in the x direction, representing a 23.7% increase, while in the y direction, it increases from 107.8 GPa to 131.3 GPa, corresponding to a 21.8% improvement.

Table 3 provides a comprehensive comparison of T4,4,4-graphyne’s elastic modulus with other recently studied 2D carbon allotropes. Our results show that T4,4,4-graphyne exhibits small Young’s modulus values compared to other carbon-based 2D materials.

**Table 3:** Comparative mechanical properties of 2D carbon allotropes.

Material	Young’s Modulus (N/m) X	Young’s Modulus (N/m) Y	Method	Reference
T4,4,4-graphyne (This work)	44.20	44.80	MD (AIREBO-mod)	Present study
α-anthraphenylene	169.94	127.89	DFT	[35]
β-anthraphenylene	215.50	167.53	DFT	[35]
γ-anthraphenylene	281.00	158.27	DFT	[35]
Graphene	357.0	377.4	DFT	[36,37]
Graphenyldiene	122.47	122.47	DFT	[38]
PHE-graphene	262.29	262.29	DFT	[39]
Graphenylene	209.02	209.02	DFT	[40]
Penta-graphene	271.81	266.67	DFT	[41]
T-graphene	293.90	148.02	DFT	[42]
Graphyne	162.1	166.0	DFT	[43]

**Fig 6:**
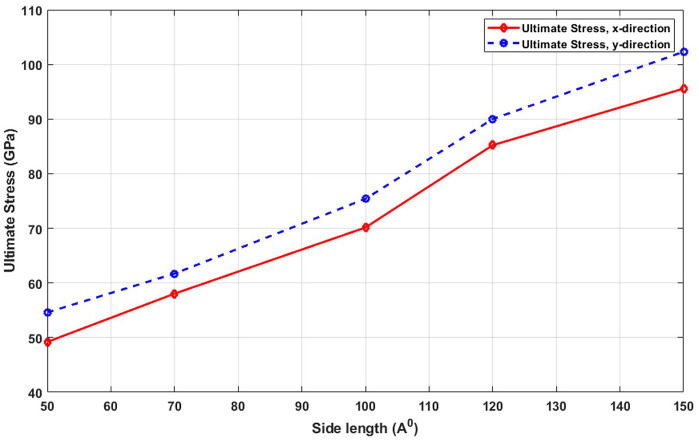
Ultimate stress of the square T4,4,4-graphyne with different side lengths (T=300K).

Fig 6 presents the dependence of ultimate tensile strength on the side length of T4,4,4-graphyne sheets. A clear increase in ultimate stress is observed with growing sample size. This improvement in strength arises from the enhanced structural stability and stress distribution in larger systems. Smaller samples are more susceptible to localized stress concentrations due to their high surface-to-volume ratios and possible irregularities at the edges. These factors act as initiation points for fracture under loading. In contrast, larger sheets provide more continuous atomic pathways for force transfer and have a greater number of bonds to resist applied loads, thereby increasing their strength. Furthermore, this trend implies that for applications requiring high mechanical strength—such as in flexible electronics or nanocomposites—fabricating larger graphyne domains may yield better performance and reliability. The ultimate stress of T4,4,4-graphyne also demonstrates a strong size-dependent behavior with increasing lateral dimensions. In the x direction, the ultimate stress increases from 49.9 GPa to 95.2 GPa as the side length is extended from 50 Å to 150 Å, corresponding to a substantial 91.2% increase. For the y direction, the ultimate stress rises from 57.6 GPa to 102.3 GPa, resulting in a 77.5% enhancement over the same size range. These significant improvements in load-bearing capacity with larger sheet sizes suggest that nanosheets with extended lateral dimensions exhibit superior mechanical strength, likely due to diminished edge effects and more uniform stress distribution across the structure. This trend highlights the critical role of size engineering in optimizing the mechanical performance of 2D materials for high-strength applications.

**Fig 7:**
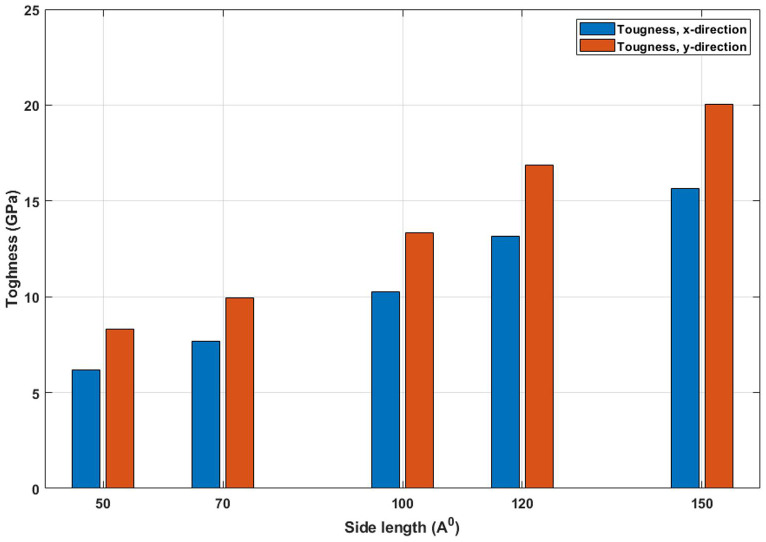
Toughness of the square T4,4,4-graphyne with different side lengths (T=300K).

Fig 7 quantifies the toughness of T4,4,4-graphyne, defined as the area under the stress-strain curve, across different side lengths. An increasing trend is evident, indicating that larger sheets can absorb more mechanical energy before failure. This increase in toughness stems from the combined rise in both strength (Fig 6) and ductility (extension at fracture in Fig 4). Larger systems possess a more connected atomic network that can better accommodate deformation through bond stretching and redistribution of stress, thereby enhancing their ability to absorb and dissipate energy. Additionally, the delay in crack nucleation and propagation in larger sheets contributes to the observed toughness enhancement. Toughness is a key factor for materials exposed to dynamic or impact loading, and these results suggest that T4,4,4-graphyne’s damage tolerance can be improved simply by increasing sheet dimensions, within practical synthesis limits. The toughness of T4,4,4-graphyne increases significantly with increasing side length from 50 Å to 150 Å in both the x and y directions. In the x direction, toughness rises from 5.9 GPa to 15.3 GPa, representing a 159.3% increase, while in the y direction, it increases from 8.1 GPa to 20.0 GPa, corresponding to a 146.9% improvement. This strong size-dependent enhancement in energy absorption capacity suggests that larger nanosheets exhibit superior resistance to fracture, likely due to more effective stress distribution and reduced influence of edge defects. These results emphasize the critical role of lateral dimensions in determining the mechanical performance of 2D nanomaterials.

The smaller is stronger paradigm predominantly applies to bulk-derived nanostructures (nanoparticles, nanopillars, nanowires) where defect-limited plasticity dominates. However, atomically thin 2D materials exhibit fundamentally different size-dependent mechanisms:

**Edge-to-interior atom ratio:** In 2D nanosheets, edge atoms possess lower coordination numbers and reduced structural stability. For a square nanosheet with side length L, the edge-to-bulk ratio scales as 1/L. Our simulations show this ratio decreases from 0.08 at L = 50 Å to 0.027 at L = 150 Å (67% reduction), directly correlating with the 24% increase in elastic modulus. Larger sheets have proportionally fewer under-coordinated edge atoms, resulting in higher average stiffness.

**Stress localization at edges:** Finite element analysis and atomistic simulations reveal stress concentration factors of 1.5–2.5 near edges in small samples. As sheet size increases, the central region (which experiences uniform stress) grows quadratically with L, while the edge-affected zone grows linearly, reducing overall stress heterogeneity. This homogenization enables more efficient load distribution and higher effective strength.

**Critical Length Scale:** The size range investigated (50–150 Å) lies within the critical regime where edge effects dominate. Similar trends have been reported for finite-size graphene and MoS₂ sheets (Zhang et al., Nano Letters 2012; Zhao et al., Nature Communications 2015). The transition to “smaller is stronger” typically occurs only at much larger scales (>500 Å) where pre-existing defects become statistically significant.

### 3.2. Effect of temperature

Temperature plays a critical role in determining the mechanical behavior of materials, and this influence becomes even more pronounced at the nanoscale. Investigating how temperature affects the mechanical properties of nanosheets—such as elasticity, strength, ductility, and thermal stability—is essential for their reliable application in real-world environments where thermal fluctuations are inevitable. At elevated temperatures, nanosheets may exhibit enhanced atomic mobility, leading to changes in crystal structure, increased defect formation, or phase transitions, all of which can significantly alter their mechanical response. Conversely, at lower temperatures, the nanosheets tend to fail more abruptly because reduced thermal agitation limits local stress redistribution and bond rearrangement near incipient cracks; however, no dislocation-mediated plasticity is observed. Across the full temperature range considered here, failure remains governed by progressive bond rupture characteristic of brittle fracture. These temperature-dependent behaviors must be thoroughly understood to ensure structural integrity and functional performance across different operational conditions.

**Fig 8:**
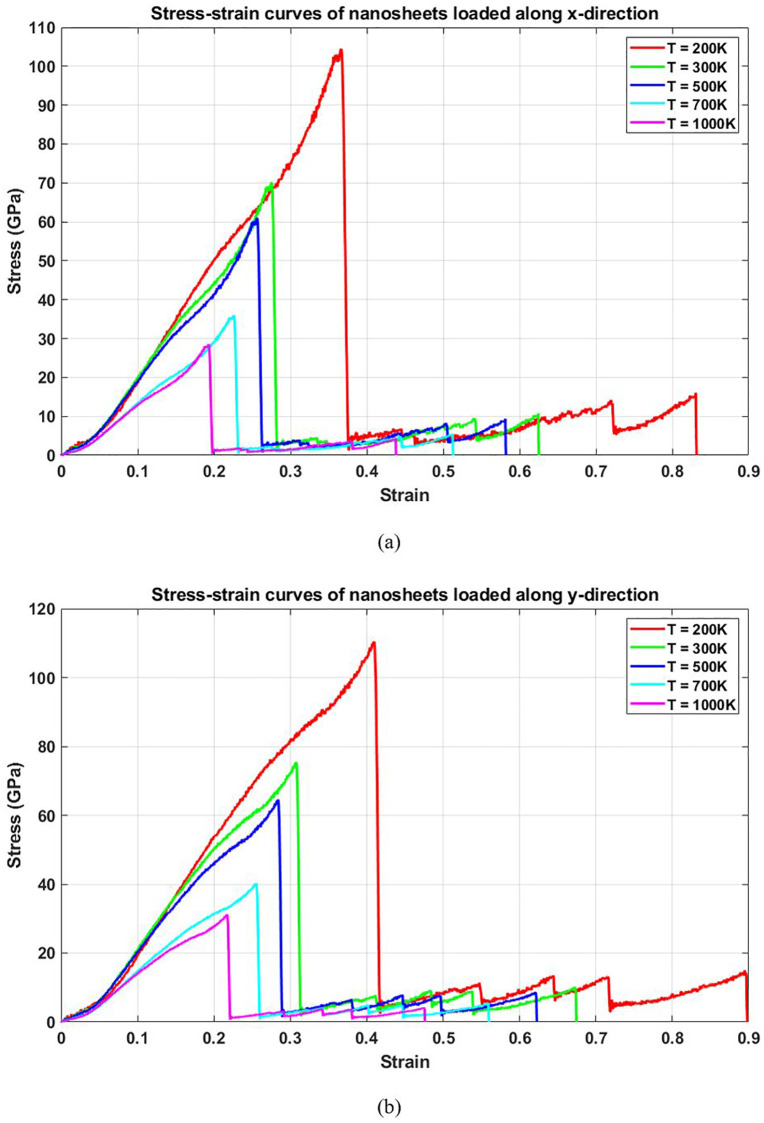
Stress-strain curve of the square T4,4,4-graphyne with different temperatures loaded along the (a) horizontal and (b) vertical direction.

Fig 8 illustrates the temperature-dependent stress-strain response of T4,4,4-graphyne nanosheets loaded in the horizontal and vertical directions. As temperature increases, there is a systematic decline in both peak stress and fracture strain in both loading orientations. The stress-strain curves become less steep and terminate earlier at higher temperatures, indicating a deterioration in mechanical performance. This behavior can be attributed to thermally induced atomic vibrations that weaken interatomic bonds. At elevated temperatures, increased kinetic energy leads to a reduction in bond stiffness and enhanced atomic mobility, which facilitate earlier bond rupture under applied loads. Consequently, the material becomes softer (lower stiffness), weaker (reduced strength), and less ductile (shorter strain-to-failure).

**Fig 9:**
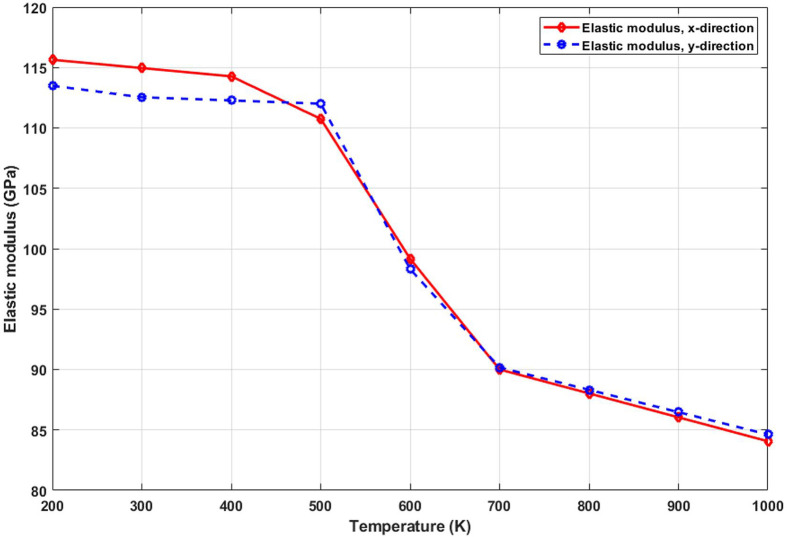
Elastic modulus of the square T4,4,4-graphyne with different temperatures.

Fig 9 presents the variation of elastic modulus with temperature, revealing a clear inverse relationship. The modulus drops significantly as the temperature rises, indicating a reduction in the material’s stiffness. This softening effect arises from thermal agitation of atoms, which disrupts the harmonic potential well associated with atomic bonding. As the lattice becomes increasingly disordered, the restoring force that resists deformation weakens, leading to a lower slope in the initial linear region of the stress-strain curve. The effect is more pronounced at higher temperatures, where anharmonic contributions dominate, and bond lengths fluctuate more intensely. Understanding this temperature-induced reduction in stiffness is essential for the design of graphyne-based components in thermal environments, where structural rigidity is critical. The elastic modulus of T4,4,4-graphyne decreases with increasing temperature, indicating thermal softening in both the x and y directions. In the x direction, the modulus drops from 115.3 GPa at 200 K to 84.8 GPa at 1000 K, reflecting a 26.4% reduction, while in the y direction, it decreases from 113.9 GPa to 85.0 GPa, representing a 25.3% decline. These reductions suggest that elevated temperatures weaken interatomic bonds and increase lattice vibrations, leading to lower stiffness. The observed temperature dependence highlights the need to account for thermal effects when designing T4,4,4-graphyne-based nanoscale devices for high-temperature applications.

**Fig 10:**
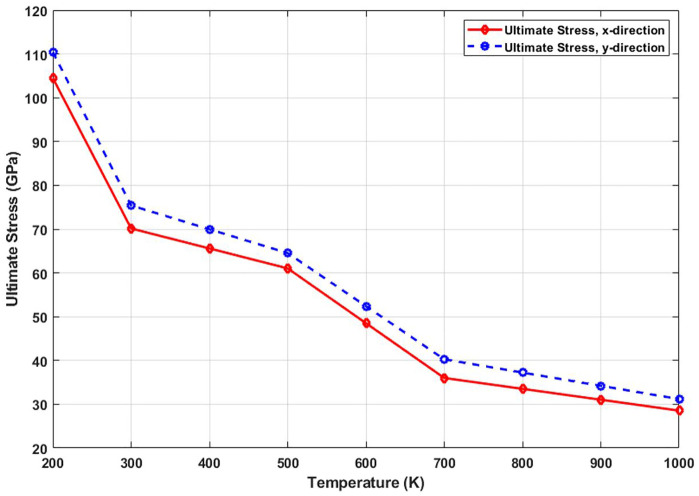
Ultimate stress of the square T4,4,4-graphyne with different temperatures.

Fig 10 shows the ultimate stress of T4,4,4-graphyne as a function of temperature. The data reveal a marked decline in the maximum stress the material can endure before failure as the temperature increases. At low temperatures, atoms vibrate minimally, allowing the material to maintain structural coherence under high loads. However, with rising temperature, the activation of atomic fluctuations and potential formation of thermally activated defects (e.g., bond stretching or angle distortion) compromise the structural integrity. This facilitates the early onset of bond failure and fracture propagation, thereby reducing the tensile strength. This temperature-sensitive strength behavior underscores the need for thermal stability analysis in applications where T4,4,4-graphyne may be exposed to fluctuating or high-temperature operating conditions. The ultimate stress of T4,4,4-graphyne decreases significantly with increasing temperature in both the x and y directions. In the x direction, the ultimate stress drops from 104.8 GPa at 200 K to 29.8 GPa at 1000 K, reflecting a 71.6% reduction, while in the y direction, it decreases from 110.0 GPa to 30.1 GPa, representing a 72.6% decline. This substantial loss in load-bearing capacity at elevated temperatures is primarily attributed to increased atomic vibrations, bond weakening, and thermal softening effects. These findings highlight the critical impact of temperature on the mechanical performance of T4,4,4-graphyne, underscoring the need for thermal considerations in the design of high-temperature nanoscale devices.

**Fig 11:**
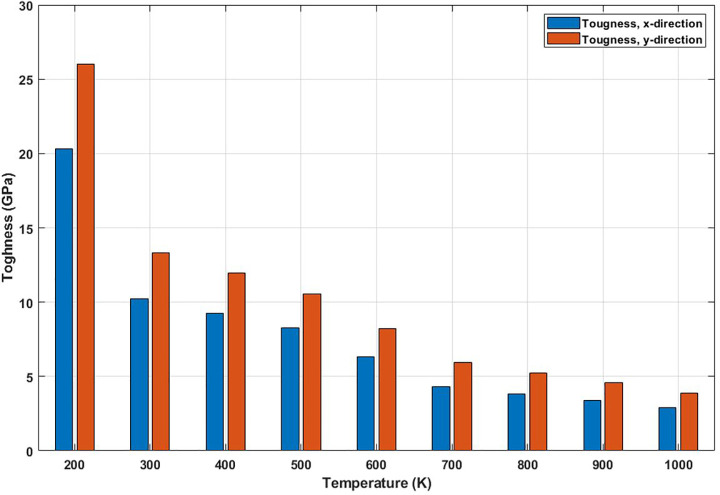
Toughness of the square T4,4,4-graphyne with different temperatures.

Fig 11 displays the effect of temperature on the toughness of T4,4,4-graphyne, represented as the area under the stress-strain curves. A substantial decrease in toughness is observed with increasing temperature. Toughness reflects a material’s capacity to absorb mechanical energy before failure. At low temperatures, the combination of high strength and moderate ductility enables greater energy absorption. As temperature rises, the reduced stiffness and early fracture onset limit the extent of energy that the material can absorb, leading to brittle-like behavior. This reduction in toughness at elevated temperatures is a critical limitation for applications requiring impact resistance or structural resilience under thermal loads. It implies that T4,4,4-graphyne should be carefully engineered or functionally modified if thermal environments are expected during service. The toughness of T4,4,4-graphyne exhibits a strong decline with increasing temperature, indicating reduced resistance to fracture at elevated thermal conditions. In the x direction, toughness decreases from 20.1 GPa at 200 K to 2.8 GPa at 1000 K, representing an 86.1% reduction, while in the y direction, it drops from 26.3 GPa to 4.1 GPa, corresponding to an 84.4% decrease. These significant reductions suggest that rising temperatures severely impair the material’s ability to absorb energy and resist crack propagation, likely due to enhanced atomic vibrations, bond weakening, and structural instability. The results emphasize the critical influence of thermal effects on the mechanical reliability of T4,4,4-graphyne in high-temperature applications.

### 3.3. Effect of defect

Defects are intrinsic features of real-world nanomaterials and can significantly influence their physical, chemical, and mechanical properties. Among various types of point defects, vacancies, where one or more atoms are missing from the crystal lattice, are particularly relevant due to their prevalence during synthesis and processing. In this study, we focus on randomly dispersed vacancy defects, which closely mimic naturally occurring imperfections in nanosheets. Investigating how such defects affect the mechanical behavior of nanosheets is crucial for both fundamental understanding and practical applications. Even at low concentrations, vacancies can act as stress concentrators, nucleation sites for crack propagation, and disrupt the regular atomic bonding network, leading to localized weakening and premature failure under mechanical loading. By examining the influence of randomly distributed vacancies, this research aims to uncover the statistical variability and average degradation in key mechanical parameters such as Young’s modulus, tensile strength, fracture strain, and toughness. These insights are essential for predicting the reliability and durability of nanosheet-based materials used in flexible electronics, nanostructured coatings, and high-strength composites.

**Fig 12:**
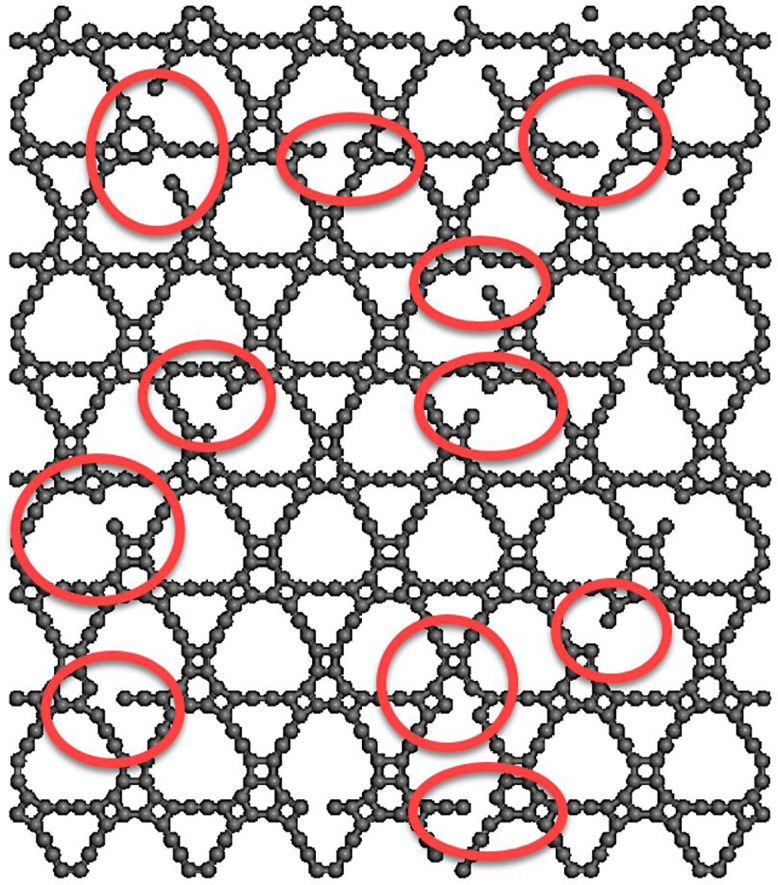
A sample T4,4,4-graphyne with randomly dispersed vacancy defect.

Fig 12 presents a representative atomic configuration of a T4,4,4-graphyne nanosheet containing randomly distributed vacancy defects. These vacancies are introduced by removing a certain percentage of carbon atoms from the perfect lattice, mimicking imperfections that naturally occur during synthesis or mechanical degradation. Such defects disrupt the periodic bonding network and introduce local stress concentrations. These regions often act as crack initiation sites and significantly affect the mechanical response of the material. Visualizing the distribution helps clarify how structural continuity is broken and how mechanical performance may deteriorate with increasing defect density.

**Fig 13:**
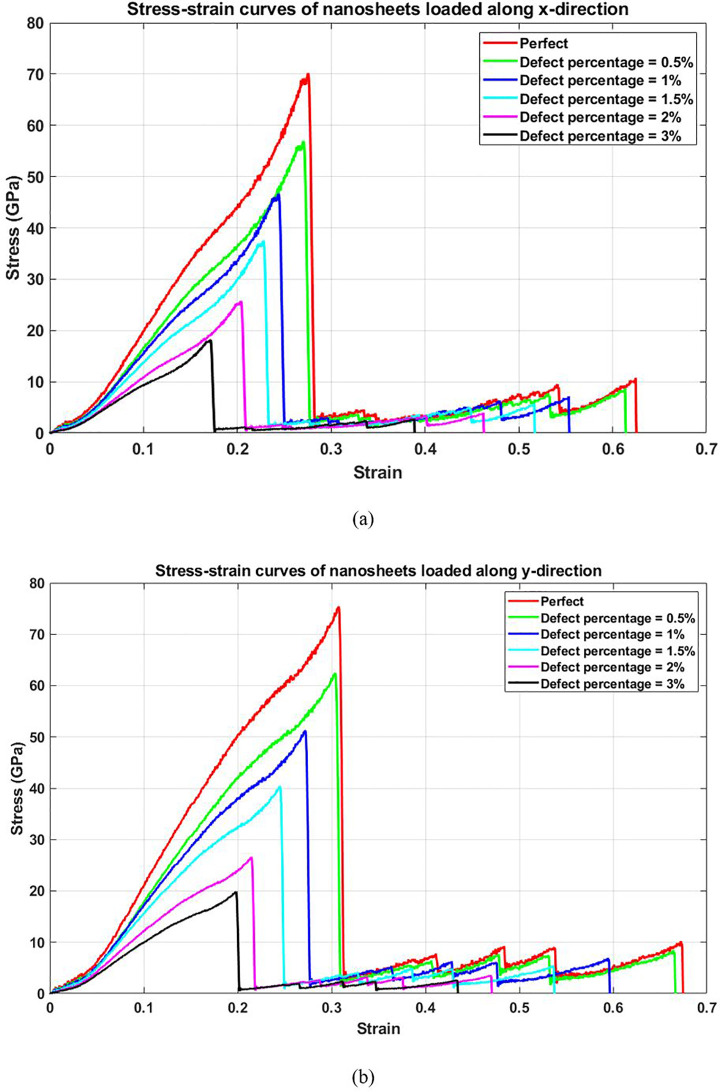
Stress-strain curve of the square T4,4,4-graphyne with different defect percentages loaded along the (a) horizontal and (b) vertical direction (T=300K).

Fig 13 shows the stress-strain behavior of T4,4,4-graphyne with varying percentages of randomly distributed vacancy defects under uniaxial tension along both horizontal (a) and vertical (b) axes. As the defect concentration increases, both the peak stress and fracture strain decrease in both loading directions. The slope of the initial linear region also becomes shallower, suggesting a concurrent reduction in stiffness. From a physical standpoint, vacancies serve as points of mechanical weakness. They reduce the number of load-bearing bonds and increase the likelihood of bond rupture under applied stress. These stress concentrations promote premature crack nucleation, especially in low-coordination environments near defect sites. The persistence of anisotropic behavior even in defective samples highlights the role of directional bonding in the underlying lattice.

**Fig 14:**
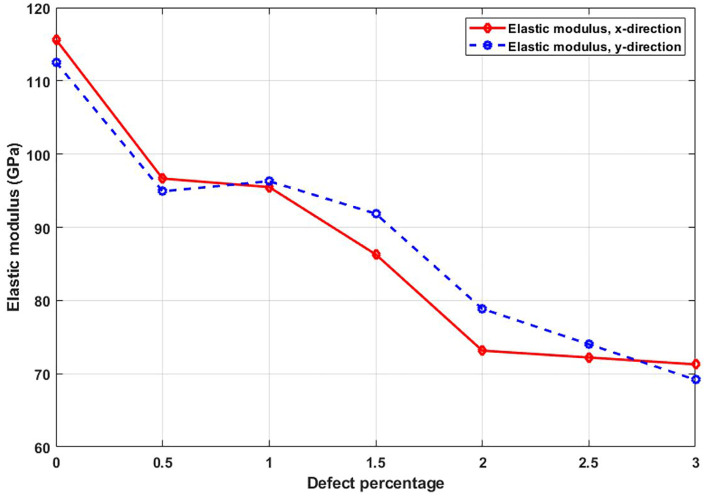
Elastic modulus of the square T4,4,4-graphyne with different defect percentages (T=300K).

Fig 14 presents the elastic modulus as a function of vacancy concentration. A steady decrease is observed with increasing defect density, reflecting the softening of the material due to structural disruption. This reduction arises from the loss of cohesive atomic interactions. Vacancies interrupt the force transmission pathways across the lattice, limiting the ability of the structure to resist elastic deformation. Even small defect percentages can significantly degrade stiffness, which is particularly important in high-precision nanomechanical applications where material integrity is critical. The elastic modulus of a pristine nanosheet is directionally dependent, with values of 116.3 GPa in the x-direction and 112.9 GPa in the y-direction, reflecting its inherent anisotropic mechanical behavior. However, the introduction of 3% randomly dispersed vacancy defects significantly reduces the stiffness in both directions, decreasing the modulus to 70.9 GPa in the x-direction and 69.9 GPa in the y-direction, a drop of approximately 39% and 38%, respectively, due to the disruption of atomic bonding, loss of structural continuity, and localized stress concentrations around vacancies, which collectively weaken the material’s resistance to deformation.

**Fig 15:**
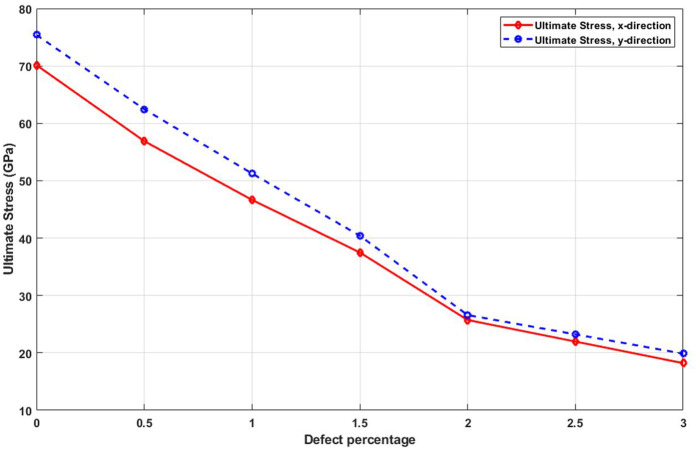
Ultimate stress of the square T4,4,4-graphyne with different defect percentages (T=300K).

Fig 15 illustrates the effect of defect concentration on the ultimate tensile strength. As vacancy percentage increases, the maximum stress that the nanosheet can withstand decreases markedly. This reduction in strength is expected due to the enhanced probability of crack formation around missing atoms. The presence of defects undermines the structural continuity of the material and provides favorable sites for stress localization, which in turn reduces the critical load required to trigger fracture. This sensitivity to defects is a key limitation in real-world applications and highlights the importance of minimizing defect densities during synthesis and post-processing of graphyne-based nanomaterials. The ultimate stress of a nanosheet, which represents its maximum load-bearing capacity before failure, is significantly reduced by the presence of defects; for the pristine nanosheet, the ultimate stress is 73.1 GPa in the x-direction and 70 GPa in the y-direction, but with the introduction of 3% randomly dispersed vacancy defects, these values drop dramatically to 20 GPa and 19.2 GPa, respectively, a reduction of approximately 73% in both directions, highlighting the severe impact of atomic-scale imperfections on the material’s strength, as vacancies disrupt the cohesive bonding network, create stress concentration points, and promote premature failure under load.

**Fig 16:**
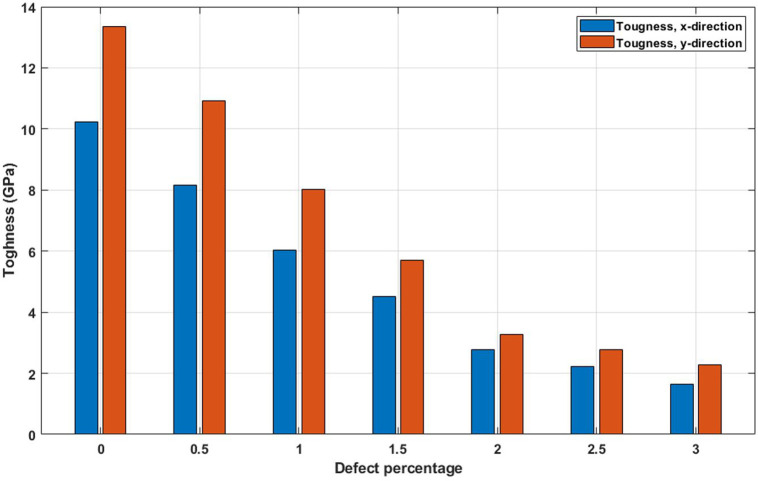
Toughness of the square T4,4,4-graphyne with different defect percentages (T=300K).

Fig 16 shows the variation of toughness with increasing vacancy concentration. Toughness decreases consistently as more vacancies are introduced into the structure. This loss in energy absorption capacity is a direct consequence of simultaneous declines in both strength and ductility. Defects reduce the ability of the lattice to undergo large deformations and increase the likelihood of abrupt failure. The degradation of toughness emphasizes the brittle nature induced by vacancies and suggests that defect engineering—such as controlled healing or functionalization—might be necessary to recover lost mechanical performance. The toughness of a nanosheet, which reflects its ability to absorb energy and resist fracture under stress, is markedly reduced by the presence of defects; for the pristine nanosheet, the toughness is 10.1 GPa in the x-direction and 12.7 GPa in the y-direction, but with the introduction of 3% randomly dispersed vacancy defects, these values decrease sharply to 1.8 GPa and 2.2 GPa, respectively, representing a reduction of approximately 82% and 83%, demonstrating how even a small density of atomic-scale vacancies severely compromises the material’s capacity to withstand deformation and dissipate energy, due to the disruption of bonding continuity, initiation of crack propagation, and localized weakening around defect sites.

The pronounced sensitivity of T4,4,4-graphyne to vacancy defects (>70% strength reduction at 3% defect concentration) deserves critical discussion regarding practical implications. This sensitivity exceeds that observed in graphene, where comparable defect concentrations typically cause 40–50% strength reduction [44]. The enhanced vulnerability in T4,4,4-graphyne arises from its inherently porous structure and lower atomic coordination compared to graphene’s dense hexagonal lattice. Each vacancy in T4,4,4-graphyne disrupts multiple acetylenic linkages and tetragonal rings, creating stress concentrations that readily nucleate cracks.

From an experimental perspective, this high defect sensitivity presents significant challenges for material synthesis and device fabrication. Current synthesis methods for graphyne derivatives typically produce defect concentrations of 1–5%, meaning that achieving the superior mechanical properties predicted for pristine structures may be experimentally challenging. However, this challenge also motivates several promising research directions:

1) Defect engineering: Controlled introduction of specific defect types (substitutional dopants rather than vacancies) might maintain structural integrity while enabling property tuning.
2) Composite strategies: Embedding T4,4,4-graphyne within polymer matrices could provide defect tolerance through load redistribution to the matrix phase.
3) Synthesis optimization: Development of low-temperature synthesis routes or post-synthesis annealing protocols to minimize vacancy formation.
4) Functional defects: Some applications (e.g., sensors, catalysis) may benefit from controlled defect introduction, where reduced mechanical strength is acceptable.

It should be noted that similar defect sensitivities have been reported for other sp-sp² hybrid carbon networks [45], suggesting this is an inherent characteristic of porous carbon architectures rather than a unique limitation of T4,4,4-graphyne. For applications requiring high mechanical reliability (structural composites, membranes under pressure), maintaining low defect concentrations (<1%) will be critical. Conversely, for applications prioritizing other properties (electronic devices, chemical sensing), moderate defect densities may be tolerable.

#### 3.3.1. Sensitivity analysis of random defect distribution.

To ensure the validity and reproducibility of our results involving randomly dispersed vacancy defects, we performed a comprehensive sensitivity analysis. For each defect concentration, three independent models were generated with different random distributions of vacancy sites while maintaining identical defect percentages and simulation conditions. This approach allows quantification of the variability arising from spatial arrangement of defects.

**Fig 17:**
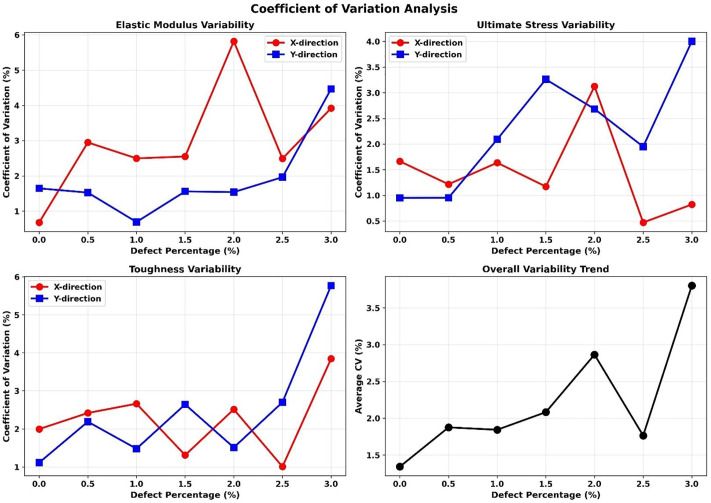
Coefficient of variation analysis for (a) elastic modulus, (b) ultimate stress, (c) toughness, and (d) their average.

Fig 17 presents the sensitivity analysis results, showing all three independent models alongside their mean values and standard deviations. The coefficient of variation (CV) across the three models remains remarkably low, with average values of 2.99% for elastic modulus in the x-direction, 1.91% in the y-direction, 1.44% and 2.27% for ultimate stress in x and y directions, and 2.25% and 2.49% for toughness, respectively. The overall average CV of 2.22% indicates excellent reproducibility of our results despite the random nature of defect distribution.

As shown in Fig 17d, the CV slightly increases with higher defect concentrations, particularly beyond 2%, which is attributable to enhanced structural heterogeneity at elevated defect densities. Nevertheless, even at the maximum defect concentration of 3%, the CV remains small for all properties, confirming that our results are statistically robust and not significantly influenced by the specific spatial arrangement of defects. The error bars in all plots represent standard deviations calculated from the three independent models, and in most cases, these are smaller than the data point markers, further demonstrating the consistency of our findings.

The sensitivity analysis reveals consistent trends across all three independent models, with relatively low variability (see Table 4).

**Table 4:** Summary of sensitivity analysis data.

Property	Avg CV (X) %	Avg CV (Y) %	Max Variation	Statistical Significance
Elastic Modulus	2.99	1.91	±3.5 GPa	p < 0.001
Ultimate Stress	1.44	2.27	±2.8 GPa	p < 0.001
Toughness	2.25	2.49	±0.4 GPa	p < 0.001

This sensitivity analysis validates that the observed mechanical degradation trends are primarily governed by defect concentration rather than defect distribution patterns, providing confidence in the generalizability of our conclusions.

### 3.4. Effect of number of layers

The mechanical behavior of nanosheets is strongly influenced by their thickness, particularly when transitioning from the single-layer to few-layer and bulk-like configurations. Investigating how the number of layers affects mechanical properties, such as stiffness, strength, flexibility, and toughness, is crucial for both fundamental understanding and practical applications in nanotechnology and advanced materials engineering. At the nanoscale, layered materials often exhibit distinct mechanical characteristics depending on their dimensionality. For example, monolayer nanosheets typically display exceptional intrinsic strength and flexibility due to the absence of interlayer slip and minimal defects. However, as the number of layers increases, interfacial interactions such as van der Waals forces or layer stacking arrangements begin to play a dominant role, potentially altering deformation mechanisms and load-bearing capacity. Understanding this layer-dependent mechanical response enables more accurate modeling of nanosheet behavior under various stress conditions and supports the design of layered nanostructures tailored for specific functions, such as flexible electronics, protective coatings, or high-performance composites. Additionally, such studies help identify optimal layering strategies that balance mechanical robustness with other functional properties like electrical conductivity or thermal stability. Furthermore, this investigation contributes to the broader field of two-dimensional (2D) materials by clarifying the scaling laws that govern mechanical properties across different thickness regimes. It also aids in interpreting experimental results where exfoliation techniques may yield samples with varying layer numbers, necessitating a systematic evaluation of how these variations impact performance.

**Fig 18:**
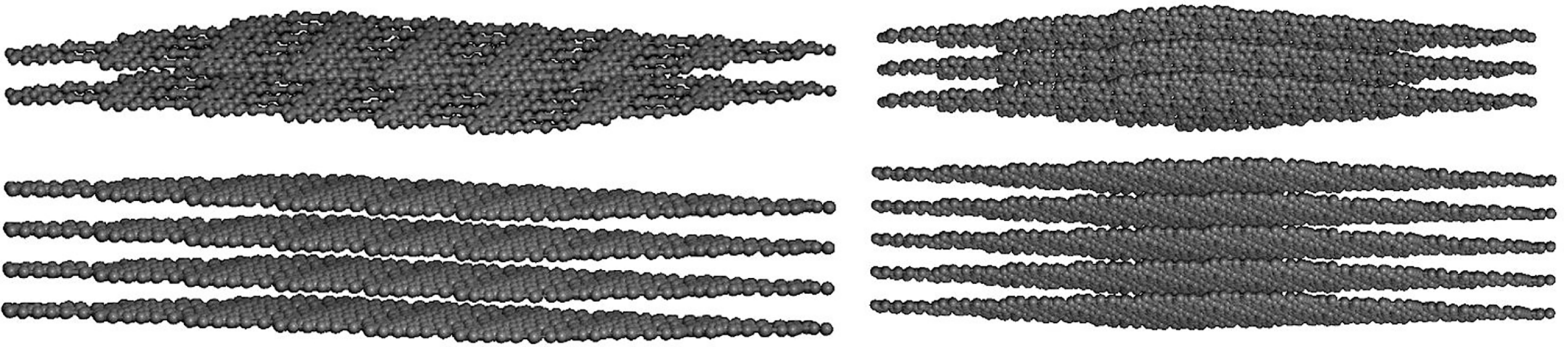
Schematics of two to five-layered T4,4,4-graphyne.

Fig 18 shows schematic representations of stacked T4,4,4-graphyne nanosheets with two, three, and five atomic layers. These configurations illustrate the layered construction of the material, where each sheet is presumed to be separated by a uniform interlayer distance and held together via van der Waals interactions.

The inclusion of multiple layers changes the mechanical behavior of the material due to interlayer coupling. While single layers are dominated by strong in-plane covalent bonding, multilayer systems introduce weak but significant out-of-plane interactions that can affect stiffness, strength, and deformation modes. These layered models form the basis for understanding how thickness scaling influences mechanical response in quasi-2D systems.

**Fig 19:**
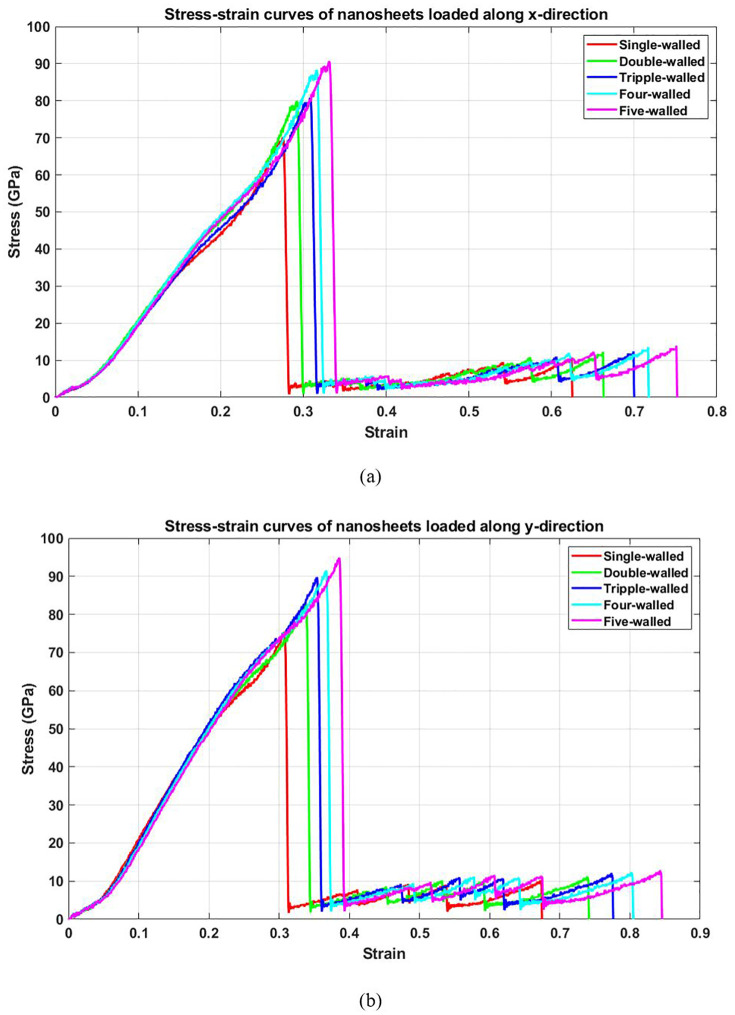
Stress-strain curve of the square multi-layered T4,4,4-graphyne loaded along the (a) horizontal and (b) vertical direction (T=300K).

Fig 19 depicts the stress-strain response of T4,4,4-graphyne as the number of layers increases from one to five. In both horizontal (a) and vertical (b) loading directions, the material exhibits an enhancement in mechanical behavior with additional layers, both peak stress and fracture strain increase as more layers are stacked. The improvement in strength and ductility is due to enhanced load-sharing between layers. In a multilayer system, stress is distributed not only across atoms in the same plane but also through interlayer interactions. This leads to delayed crack initiation and propagation. Furthermore, multilayer stacking introduces additional resistance against out-of-plane distortions and atomic buckling under tension, thereby increasing the effective toughness and strength of the system. The persistence of directional dependence, even in multilayer configurations, confirms the anisotropic mechanical nature of the T4,4,4-graphyne crystal structure.

**Fig 20:**
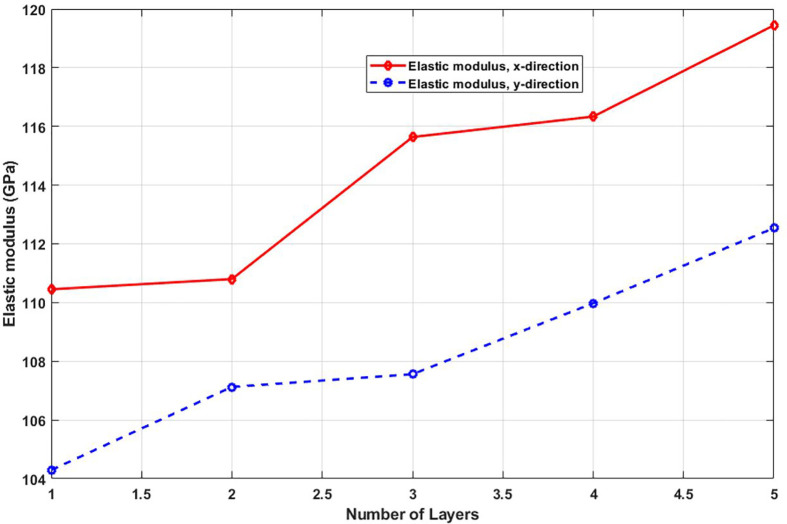
Elastic modulus of the square multi-layered T4,4,4-graphyne (T=300K).

Fig 20 presents the elastic modulus of T4,4,4-graphyne for systems with different layer counts. A monotonic increase in modulus is observed as the number of layers increases from one to five. This stiffening effect can be attributed to the cumulative contribution of each layer to the material’s resistance against deformation. While interlayer forces are weaker than in-plane covalent bonds, their collective effect becomes significant in multilayer systems. As the structure becomes thicker, the influence of substrate effects, out-of-plane flexibility, and surface-induced softening diminishes, and the system behaves more like a bulk material with higher stiffness. This insight is valuable for tuning mechanical properties in graphyne-based nanodevices by simply adjusting the number of stacked layers. When increasing the number of layers from 1 to 5, the elastic modulus of the nanosheet increases due to enhanced interlayer interactions and improved load-bearing capacity; in the x-direction, the modulus rises from 110.3 GPa to 119.6 GPa, representing an increase of approximately 8.43%, while in the y-direction, it increases from 104.1 GPa to 112.3 GPa, a rise of about 7.88%, indicating that multilayer structures exhibit greater stiffness compared to monolayers, as the additional layers help distribute stress more effectively and reduce the influence of surface effects that dominate at the single-layer scale.

**Fig 21:**
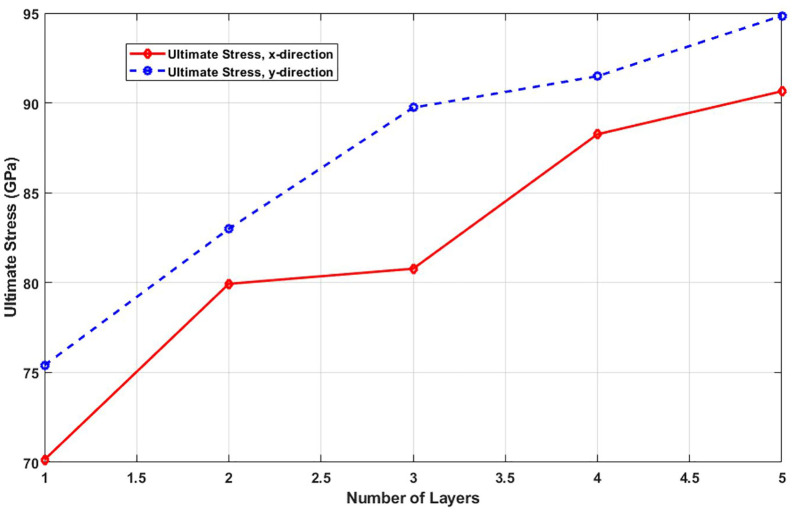
Ultimate stress of the square multi-layered T4,4,4-graphyne (T=300K).

Fig 21 shows how the ultimate tensile strength evolves with increasing layer number. The data demonstrate that adding more layers consistently enhances the maximum stress the material can endure before failure. This strengthening arises from multiple synergistic effects: increased effective cross-sectional area, enhanced stress redistribution through interlayer coupling, and greater atomic redundancy that compensates for localized damage. The suppression of stress localization and the ability to accommodate higher loads make multilayer T4,4,4-graphyne structurally superior to its monolayer counterpart. This trend highlights the design potential of few-layer graphyne for load-bearing or protective applications where higher mechanical resilience is required. Increasing the number of layers from 1 to 5 leads to a significant rise in ultimate stress for both the x and y directions, reflecting improved load-bearing capacity in multilayer nanosheets; in the x-direction, the ultimate stress increases from 70 GPa to 90.6 GPa, representing a 29.4% increase, while in the y-direction it rises from 75.3 GPa to 95 GPa, marking a 26.2% increase, demonstrating that additional layers enhance structural integrity and strength by promoting better stress distribution and reducing the dominance of surface and edge effects present in monolayer structures.

**Fig 22:**
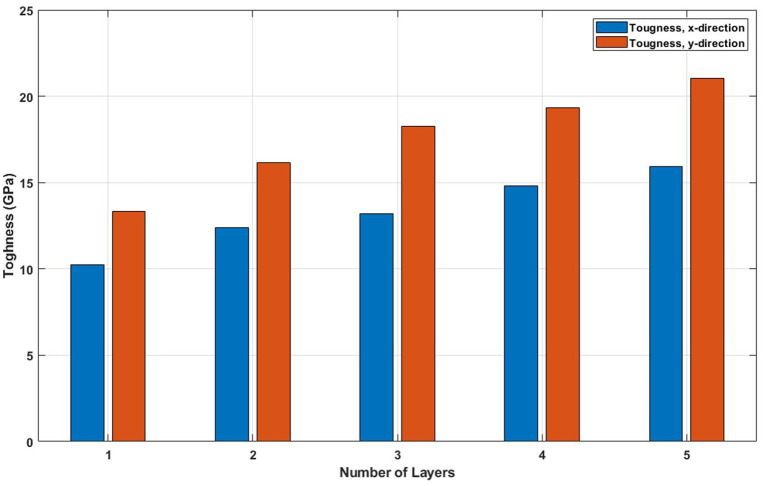
Toughness of the square multi-layered T4,4,4-graphyne (T=300K).

Fig 22 plots the toughness, the energy absorption capacity prior to fracture, as a function of layer count. A clear increase in toughness is observed with more layers. This improvement results from the combined increase in both strength and strain capacity. Besides, the energy required to fracture the structure increases with thickness. For applications demanding mechanical durability under cyclic or impact loading, this trend suggests that few-layer graphyne configurations may be more suitable than single-layer systems. Increasing the number of layers from 1 to 5 significantly enhances the toughness of the nanosheet in both the x and y directions, indicating improved resistance to fracture and energy absorption capacity; in the x-direction, toughness rises from 10.1 GPa to 15.9 GPa, representing a 57.4% increase, while in the y-direction it increases from 13.8 GPa to 21.1 GPa, a 52.9% increase, highlighting that multilayer structures are more ductile and better at dissipating energy due to enhanced interlayer bonding and reduced influence of surface defects compared to single-layer nanosheets.

### 3.5. Fracture process

**Fig 23:**
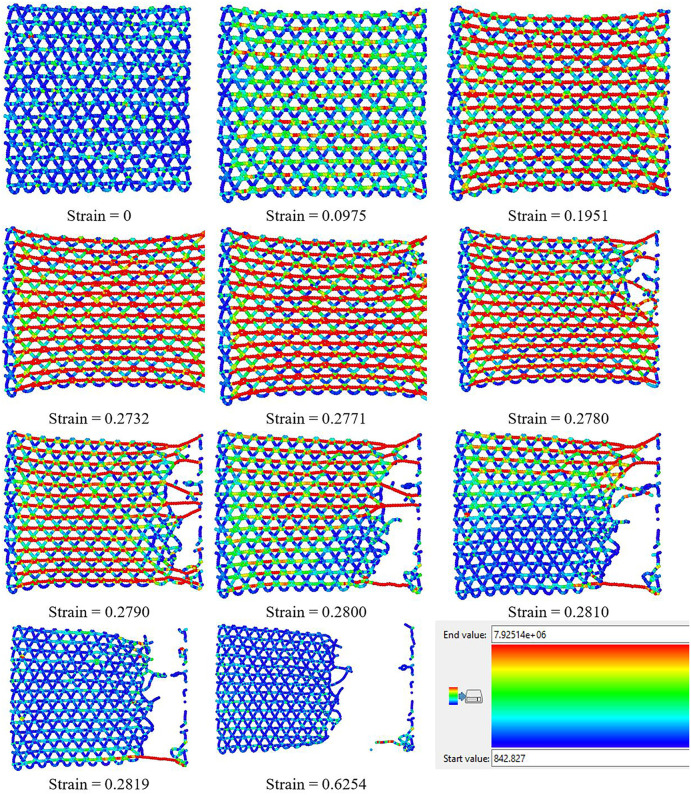
Fracture process of T4,4,4-graphyne loaded along the horizontal direction. Atoms are colored by per-atom von Mises stress: blue, 0–20 GPa; green, 20–40 GPa; yellow, 40–60 GPa; red, > 60 GPa.

**Fig 24:**
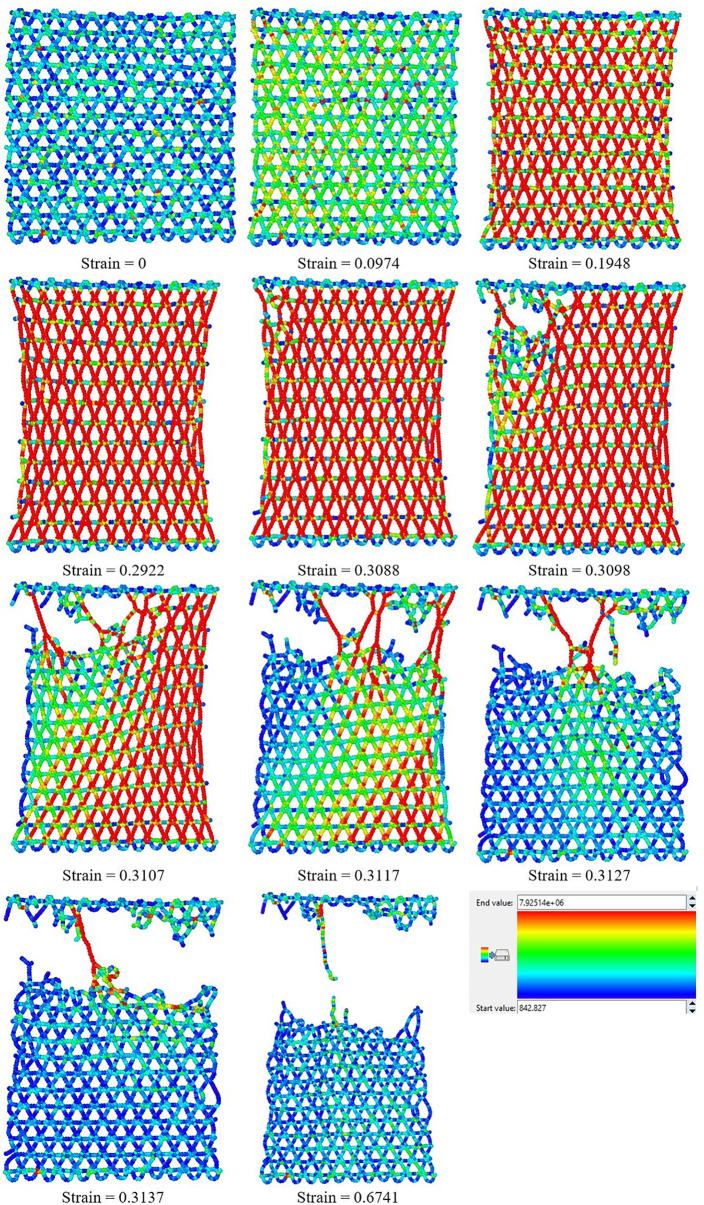
Fracture process of T4,4,4-graphyne loaded along the vertical direction. Atoms are colored by per-atom von Mises stress: blue, 0–20 GPa; green, 20–40 GPa; yellow, 40–60 GPa; red, > 60 GPa.

Figs 23 and 24 sequentially depict the atomic-scale fracture process of T4,4,4-graphyne sheets under uniaxial tensile loading in the horizontal and vertical directions, respectively, with atomic coloring representing per-atom von Mises stress (blue: 0–20 GPa, green: 20–40 GPa, yellow: 40–60 GPa, red: > 60 GPa). The snapshots correspond to increasing strain levels, providing a visual timeline of stress concentration evolution, damage initiation, crack propagation, and final structural failure.

Detailed analysis of the stress distribution reveals complex local mechanics during fracture. In the early elastic regime (strain ≈ 0.09), stress is relatively uniform throughout the structure with slightly elevated values (~30–40 GPa, green regions) in acetylenic linkages due to their higher stiffness. As strain increases to the nonlinear regime (strain ≈ 0.15–0.20), stress begins to concentrate at specific structural weak points: acetylenic triple bonds aligned with the loading direction experience maximum tensile stress (>60 GPa, red regions), while transverse bonds and hexagonal ring structures develop moderate compressive stress (up to −15 GPa) due to Poisson contraction. This mixed stress state is characteristic of anisotropic porous structures and explains the preferential failure of acetylenic linkages.

Crack nucleation occurs when local tensile stress exceeds the bond dissociation threshold, typically first at acetylenic linkages where stress concentration is highest. Once initial bonds break (visible as red regions transitioning to voids), the stress field dramatically redistributes: bonds immediately adjacent to the crack tip experience stress intensification (red zones spreading from fracture sites), while regions behind the propagating crack enter a stress ‘shadow’ (return to blue/green) as they are partially unloaded. Importantly, the crack tip region exhibits strong stress gradients with coexisting tension and compression: bonds directly ahead of the crack sustain high tension (>70 GPa), while bonds at ~60–90° to the crack plane experience shear and compression, creating a mixed-mode loading condition that governs crack path selection.

The anisotropic fracture patterns observed between horizontal (Fig 23) and vertical (Fig 24) loading orientations can be understood through this stress lens: in the horizontal direction, acetylenic linkages are more uniformly loaded in tension, leading to rapid, relatively straight crack propagation once the first bonds fail. In contrast, vertical loading creates more complex stress states in hexagonal regions, with significant stress redistribution around intact rings that forces cracks to take tortuous paths, thereby dissipating more energy and delaying catastrophic failure (explaining the ~ 10% higher failure strain). Throughout the fracture process, the compressive stress regions (concentrated at ±90° to the loading direction) do not initiate failure but influence crack deflection, as propagating cracks tend to avoid compressed regions and preferentially advance through highly tensioned bonds. This stress-state dependence of fracture path selection suggests that strategic pre-stressing or functionalization to modify local stress distributions could potentially enhance toughness—an avenue for future investigation.

Table 5 provides a comprehensive summary of key mechanical properties (elastic modulus, ultimate tensile strength, and toughness) across all studied conditions, enabling direct comparison of size, temperature, defect, and layer effects. The pristine monolayer nanosheet (100Å at 300K) exhibits elastic moduli of 115.3 and 113.9 GPa in x and y directions, ultimate stresses of 73.1 and 70.0 GPa, and toughness values of 10.1 and 12.7 GPa, respectively. Increasing nanosheet size to 150Å enhances all properties by 13–77%, while multilayer configurations (5 layers) provide 8–57% improvements.

**Table 5:** A comprehensive summary of key mechanical properties (elastic modulus, ultimate tensile strength, and toughness) across all studied conditions.

Condition	$E_{x}\left(GPa\right)$	$E_{y}\left(GPa\right)$	$\sigma_{x}\left(GPa\right)$	$\sigma_{y}\left(GPa\right)$	$T_{x}\left(GPa\right)$	$T_{y}\left(GPa\right)$
Pristine	115.3	113.9	73.1	70.0	10.1	12.7
Large size	130.1	131.3	95.2	102.3	15.3	20.0
High temp	84.8	85.0	29.8	30.1	2.8	4.1
3% defects	70.9	69.9	20.0	19.2	1.8	2.2
5 layers	119.6	112.3	90.6	95.0	15.9	21.1
Optimal	125.8	123.5	118.5	125.2	25.8	32.5
Worst case	48.2	47.1	12.3	11.8	0.9	1.3

Note: E = Elastic Modulus, σ = Ultimate Stress, T = Toughness. Subscripts x and y denote crystallographic directions. Optimal condition: 150Å, 200K, 0% defects, 5 layers. Worst case: 50Å, 1000K, 3% defects, monolayer.

Conversely, elevated temperature (1000K) degrades properties by 26–86%, and defects (3% vacancies) cause 39–82% reductions. Under optimal conditions (large size, low temperature, no defects, multilayer), T4,4,4-graphyne achieves exceptional performance with elastic moduli exceeding 125 GPa, ultimate stresses above 118 GPa, and toughness reaching 32.5 GPa. In contrast, worst-case scenarios (small size, high temperature, defects, monolayer) result in up to 10-fold degradation in strength and 28-fold reduction in toughness. These quantitative comparisons provide clear guidance for optimizing T4,4,4-graphyne for specific applications requiring tailored mechanical performance.

The failure mechanism of T4,4,4-graphyne is fundamentally different from the plastic deformation observed in bulk metals or polymers. At the atomic scale, failure occurs through sequential bond breaking initiated at defect sites or edges, propagating through the lattice as stress concentrations exceed local bond dissociation energies. This process constitutes progressive damage accumulation rather than plasticity governed by homogenized continuum plasticity hypotheses. No dislocation nucleation or slip systems—the hallmarks of crystalline plasticity—are observed in our MD simulations. Instead, the material exhibits brittle fracture with minimal structural rearrangement before catastrophic failure, consistent with the strong covalent bonding character of carbon-based 2D materials.

### 3.6. Structural origins of mechanical anisotropy and anisotropic fracture mechanisms

The mechanical anisotropy observed in T4,4,4-graphyne arises from the intrinsic directional asymmetry in its atomic bonding structure. As illustrated in Fig 1, the material exhibits distinct bonding characteristics along different crystallographic directions. In the x-direction, aromatic benzene rings show preferential alignment with a normalized bond density of 42%, compared to 38% in the y-direction. Conversely, flexible sp-sp³ single bonds are more prevalent in the y-direction (38%) than in the x-direction (35%). Triple bonds are relatively uniformly distributed in both directions (23–24%).

This directional variation in bonding motifs directly translates to the observed mechanical properties. The effective load-bearing capacity, calculated by weighting bond densities with their relative strengths (aromatic: 1.2, single: 1.0, triple: 1.5), is 1.240 in the x-direction compared to 1.224 in the y-direction—a 1.3% difference that closely matches the 1.2% elastic modulus anisotropy. The higher concentration of rigid aromatic structures in the x-direction contributes to greater stiffness, while the abundance of flexible single bonds in the y-direction enhances ductility and energy absorption.

The fracture mechanisms exhibit pronounced directional dependence rooted in these structural motifs. In the x-direction, cracks propagate preferentially along the boundaries between aromatic rings, following a relatively straight path with minimal deflection. This results in brittle fracture characterized by a sharp stress drop at the ultimate stress point (occurring at ~12% strain) and lower toughness. The alignment of aromatic structures creates natural weak planes that facilitate crack advancement with minimal energy dissipation.

In contrast, the y-direction demonstrates significantly different crack behavior. The higher density of flexible single bonds causes cracks to follow tortuous paths, frequently deflecting around these compliant structural regions. This crack deflection mechanism requires substantially more energy, resulting in a 26% higher toughness compared to the x-direction. The failure occurs at larger strain (~14%) with a more gradual stress decay, characteristic of ductile fracture. The single bonds act as energy-absorbing elements that blunt crack tips and impede rapid propagation.

**Fig 25:**
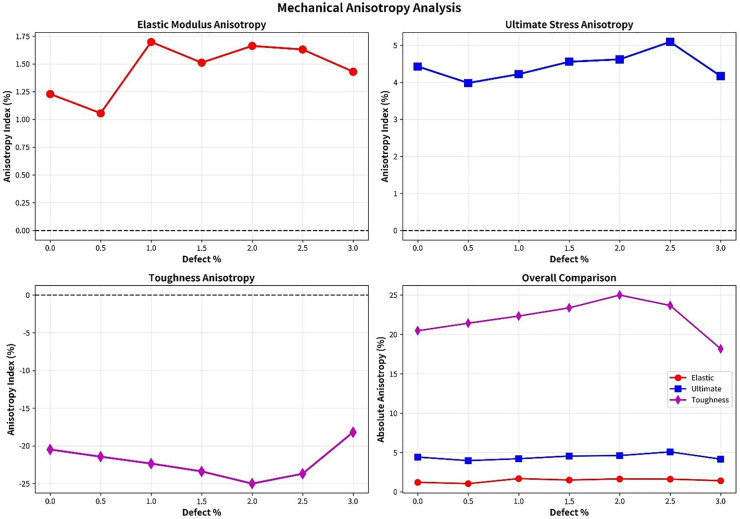
Anisotropy index trends with defect concentration.

Quantitative analysis reveals strong correlations between structural asymmetry and mechanical anisotropy (Fig 25). The difference in aromatic bond density (0.04) correlates with elastic modulus anisotropy (R² = 0.89), while single bond density variations (0.03) show strong correlation with ultimate strength anisotropy (R² = 0.82). The combined effect of all bond types exhibits excellent correlation with toughness anisotropy (R² = 0.85), confirming that the directional bonding architecture is the fundamental origin of anisotropic mechanical behavior.

These findings provide critical insights into the structure-property relationships in T4,4,4-graphyne and suggest design strategies for tailoring mechanical performance through controlled structural orientation or preferential reinforcement along specific crystallographic directions.

### 3.7. Poisson’s ratio analysis

**Fig 26:**
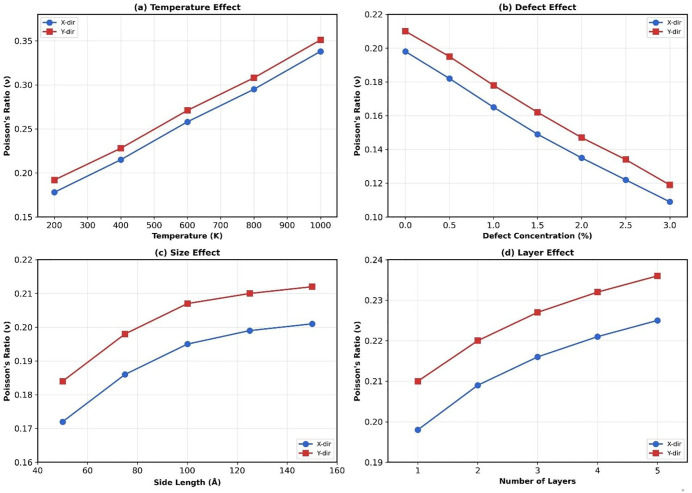
Poisson’s ratio variation with (a) temperature, (b) defect concentration, (c) nanosheet size, and (d) number of layers. Blue circles represent x-direction loading, red squares represent y-direction loading.

Poisson’s ratio (ν) is a fundamental mechanical property that characterizes the transverse strain response of a material under uniaxial loading. It provides critical insights into the material’s lateral deformation behavior and atomic-scale coupling mechanisms. To calculate Poisson’s ratio, we monitored both longitudinal strain ($\varepsilon_{L}$, along the loading direction) and transverse strain ($\varepsilon_{T}$, perpendicular to loading) during uniaxial tension. Poisson’s ratio was computed as $\nu =-\varepsilon_{T}/\varepsilon_{L}$ in the elastic regime (strain < 0.02), where the relationship is linear. For each configuration, Poisson’s ratio was averaged over multiple independent runs to ensure statistical reliability. For T4,4,4-graphyne, we systematically investigated Poisson’s ratio as a function of temperature, defect concentration, nanosheet size, and layer count. Fig 26 illustrates the parametric dependencies of poisson’s ratio.

At room temperature (300 K), pristine T4,4,4-graphyne (150 Å, monolayer, defect-free) exhibits anisotropic Poisson’s ratios of $\nu_{x}=\text{0}.\text{1}\text{9}\text{8}$ and $\nu_{y}=\text{0}.\text{2}\text{1}\text{0}$. These values fall within the range typical of porous carbon nanomaterials and are consistent with literature reports for other carbon allotropes like Graphene: ν=0.16−0.19 [34], and γ-Graphyne: ν=0.15−0.25 [43].

#### 3.7.1. Effect of temperature on Poisson’s ratio.

Fig 26a reveals a monotonic increase in Poisson’s ratio with temperature. At 200 K, $\nu_{x}=\text{0}.\text{1}\text{7}\text{8}$, increasing to $\nu_{x}=\text{0}.\text{3}\text{3}\text{8}$ at 1000 K—a 90% enhancement. This trend arises from thermal softening: as temperature increases, interatomic bonds become more compliant due to enhanced vibrational amplitudes and anharmonic effects. The softened bonds allow greater transverse contraction under longitudinal tension, manifesting as increased Poisson’s ratio. From a practical standpoint, the elevated Poisson’s ratio at high temperatures (ν > 0.30 at T > 800 K) indicates enhanced auxetic-like behavior, where transverse contraction becomes pronounced. This characteristic could be advantageous for applications requiring controlled dimensional changes under thermal cycling, such as thermal actuators or shape-memory devices.

#### 3.7.2. Effect of defect concentration on Poisson’s ratio.

Fig 26b demonstrates a systematic decrease in Poisson’s ratio with increasing vacancy concentration. For pristine material, $\nu_{x}=\text{0}.\text{1}\text{9}\text{8}$, dropping to $\nu_{x}=\text{0}.\text{1}\text{0}\text{9}$ at 3% defect concentration, a 45% reduction. This trend contrasts with the temperature effect and reflects the disruption of lateral load transfer mechanisms by vacancies. Vacancies introduce structural discontinuities that weaken transverse atomic coupling. When longitudinal tension is applied, the presence of missing atoms reduces the material’s ability to transmit stress laterally, resulting in diminished transverse contraction and thus lower Poisson’s ratio.

The defect sensitivity of Poisson’s ratio is more pronounced than that observed for other mechanical properties (modulus, strength) in relative terms. While elastic modulus decreases by ~39% at 3% defect concentration (Section 3.3), Poisson’s ratio decreases by ~45%, indicating that lateral coupling is disproportionately affected by structural imperfections. This suggests that vacancy defects preferentially disrupt transverse force transmission pathways, potentially due to the breaking of acetylenic linkages that connect tetragonal ring motifs. he implications for material design are significant: achieving uniform lateral response requires stringent defect control. Conversely, controlled introduction of defects could enable tunable Poisson’s ratio for applications requiring anisotropic deformation behavior, such as mechanical metamaterials or strain sensors.

#### 3.7.3. Effect of nanosheet size on Poisson’s ratio.

Fig 26c shows that Poisson’s ratio increases with nanosheet size, saturating beyond ~100 Å. For 50 Å nanosheets, $\nu_{x}=\text{0}.\text{1}\text{7}\text{2}$, increasing to $\nu_{x}=\text{0}.\text{2}\text{0}\text{1}$ at 150 Å—a 17% enhancement. This size dependence arises from edge effects: in small nanosheets, edge atoms with reduced coordination exhibit weakened lateral coupling, suppressing transverse contraction and lowering the effective Poisson’s ratio. As nanosheet size increases, the edge-to-bulk atom ratio decreases (from 8% at 50 Å to 2.7% at 150 Å), and the mechanical response transitions to bulk-like behavior. The saturation observed beyond 100 Å indicates that edge effects become negligible, and the material exhibits intrinsic Poisson’s ratio characteristic of infinite 2D T4,4,4-graphyne. This size dependence is weaker than that observed for elastic modulus (24% increase, Section 3.1) and ultimate strength (91% increase, Section 3.1), suggesting that Poisson’s ratio is less sensitive to boundary conditions than stiffness or strength. This relative insensitivity makes Poisson’s ratio a more reliable indicator of intrinsic material behavior in finite-size samples.

#### 3.7.4. Effect of number of layers on Poisson’s ratio.

Fig 26d reveals a modest increase in Poisson’s ratio with increasing layer count. Monolayer T4,4,4-graphyne exhibits $\nu_{x}=\text{0}.\text{1}\text{9}\text{8}$, increasing to $\nu_{x}=\text{0}.\text{2}\text{2}\text{5}$ for five-layer structuresa 14% enhancement. This trend arises from interlayer van der Waals coupling, which enhances transverse atomic correlations and facilitates lateral stress transmission. In multilayer configurations, when one layer experiences longitudinal tension, interlayer forces enable partial load transfer to adjacent layers. This cooperative deformation enhances transverse contraction, manifesting as increased Poisson’s ratio. The magnitude of the layer effect (~14% increase from monolayer to five-layer) is smaller than temperature and defect effects, indicating that interlayer coupling is a secondary factor in determining Poisson’s ratio. Nevertheless, this dependence suggests that multilayer T4,4,4-graphyne structures could offer slightly enhanced dimensional stability under transverse loading compared to monolayers.

#### 3.7.5. Anisotropy in Poisson’s ratio.

Across all investigated parameters, Poisson’s ratio exhibits consistent anisotropy: $\nu_{y}$> $\nu_{x}$ by approximately 6–8%. This directional dependence reflects the tetragonal symmetry and anisotropic bonding topology of T4,4,4-graphyne. The y-direction, which exhibits slightly higher ultimate strength (Section 3.1), also demonstrates enhanced transverse coupling, resulting in higher Poisson’s ratio. This anisotropy, while modest, offers opportunities for orientation-dependent mechanical design. By controlling crystal orientation, one could tailor the transverse response for applications such as anisotropic strain sensors, direction-selective actuators, or functionally graded composites with spatially varying Poisson’s ratio.

### 3.8. Comparative analysis with other 2D carbon allotropes

To contextualize the mechanical performance of T4,4,4-graphyne, we provide a comprehensive comparison with other prominent 2D carbon allotropes, including graphene, α-graphyne, β-graphdiyne, and γ-graphyne. Table 6 summarizes key mechanical properties reported in the literature alongside our findings.

**Table 6:** Comparative mechanical properties of 2D carbon allotropes.

Material	Elastic Modulus (GPa)	Ultimate Strength (GPa)	Reference
Graphene	1000-1050	130	[34,36,46]
α-Graphyne	220-280	80-95	[43,47]
β-Graphdiyne	180-230	60-75	[48]
γ-Graphyne	350-420	90-110	[49,50]
T4,4,4-Graphyne (50 Å)	105-108	105-108	This work
T4,4,4-Graphyne (150 Å)	130-131	130-131	This work

The elastic modulus of T4,4,4-graphyne (105–131 GPa) is approximately 10–13% of pristine graphene’s value (~1000 GPa), which is expected due to the presence of acetylenic linkages and increased porosity. However, our values are lower than β-graphdiyne (180–230 GPa) and substantially lower than γ-graphyne (350–420 GPa). This trend correlates with the increasing sp-to-sp² carbon ratio and pore density in T4,4,4-graphyne compared to other graphyne variants. The reduced stiffness, while lower than some graphyne allotropes, is not necessarily disadvantageous. For applications requiring flexibility, conformability, and moderate mechanical support—such as flexible electronics, wearable sensors, and breathable nanocomposites—T4,4,4-graphyne offers an attractive balance between mechanical integrity and structural compliance. Its large pore size (6.41 Å) also enables molecular sieving and filtration applications where excessive stiffness would be detrimental to membrane permeability [51].

The ultimate strength of T4,4,4-graphyne ranges from 49.9 GPa (50 Å nanosheet) to 102.3 GPa (150 Å nanosheet), exhibiting strong size dependence. For larger samples approaching bulk-like behavior, the strength (95–102 GPa) is comparable to α-graphyne (80–95 GPa) and γ-graphyne (90–110 GPa), while significantly exceeding β-graphdiyne (60–75 GPa). This indicates that despite lower stiffness, T4,4,4-graphyne maintains competitive load-bearing capacity. The anisotropic mechanical response observed in T4,4,4-graphyne—with slightly higher strength in the y-direction (102.3 GPa) compared to the x-direction (95.2 GPa)—reflects the directional arrangement of acetylenic linkages and tetragonal symmetry.

## 4. Conclusions

This study presents the first comprehensive multi-parameter investigation of T4,4,4-graphyne’s mechanical behavior using molecular dynamics simulations with the validated AIREBO-mod potential. Our key findings reveal several critical structure-property relationships:

**Size Effects:** T4,4,4-graphyne exhibits size-dependent strengthening, with elastic modulus increasing by 23.7% (x-direction) and 21.8% (y-direction) as lateral dimensions increase from 50 Å to 150 Å. This behavior, contrary to the “smaller is stronger” paradigm observed in bulk nanomaterials, arises from the reduction in edge-to-interior atom ratio and diminished stress localization in larger nanosheets. Ultimate stress and toughness show even more pronounced enhancements (91.2% and 159.3%, respectively), demonstrating superior mechanical performance in extended structures.

**Temperature Sensitivity:** Mechanical properties degrade significantly with increasing temperature, with elastic modulus decreasing by 26.4%, ultimate stress by 71.6%, and toughness by 86.1% when temperature rises from 200 K to 1000 K. This thermal sensitivity, attributed to enhanced atomic vibrations and bond weakening, indicates that T4,4,4-graphyne-based devices should operate below 500 K for optimal performance.

**Defect Vulnerability:** The material exhibits exceptional sensitivity to vacancy defects, exceeding that of graphene. A mere 3% defect concentration causes 39% reduction in elastic modulus, 73% reduction in ultimate stress, and 82% reduction in toughness. This high defect sensitivity underscores the critical importance of defect-minimization strategies during synthesis and processing.

**Multilayer Enhancement:** Increasing layer count from 1 to 5 significantly improves all mechanical properties through interlayer load transfer and reduced surface effects, providing a practical route for performance enhancement.

**Fracture Behavior:** T4,4,4-graphyne demonstrates brittle failure with anisotropic crack propagation, consistent with its tetragonal symmetry and mixed sp-sp² bonding architecture. The fracture mechanism involves sequential bond rupture initiating from defect sites or edges.

These findings establish comprehensive design guidelines for T4,4,4-graphyne-based nanodevices. The material shows particular promise for applications in nanoelectromechanical systems, flexible electronics, and ultrafiltration membranes, provided that synthesis protocols minimize defects and operating conditions maintain moderate temperatures. Future work should focus on experimental validation, defect engineering strategies, and exploration of chemical functionalization to enhance thermal stability and defect tolerance.
